# Integrated spatial multi‐omics profiling of *Fusobacterium nucleatum* in breast cancer unveils its role in tumour microenvironment modulation and cancer progression

**DOI:** 10.1002/ctm2.70273

**Published:** 2025-03-11

**Authors:** Feng Zhao, Rui An, Yilei Ma, Shaobo Yu, Yuzhen Gao, Yanzhong Wang, Haitao Yu, Xinyou Xie, Jun Zhang

**Affiliations:** ^1^ Department of Clinical Laboratory Sir Run Run Shaw Hospital Zhejiang University School of Medicine Hangzhou Zhejiang People's Republic of China; ^2^ Key Laboratory of Precision Medicine in Diagnosis and Monitoring Research of Zhejiang Province Hangzhou Zhejiang People's Republic of China

**Keywords:** breast cancer, *Fusobacterium nucleatum*, intratumoral microbiota, spatial multi‐omics, tumour microenvironment

## Abstract

Tumour‐associated microbiota are integral components of the tumour microenvironment (TME). However, previous studies on intratumoral microbiota primarily rely on bulk tissue analysis, which may obscure their spatial distribution and localized effects. In this study, we applied in situ spatial‐profiling technology to investigate the spatial distribution of intratumoral microbiota in breast cancer and their interactions with the local TME. Using 5R 16S rRNA gene sequencing and RNAscope FISH/CISH on patients’ tissue, we identified significant spatial heterogeneity in intratumoral microbiota, with *Fusobacterium nucleatum* (*F. nucleatum*) predominantly localized in tumour cell‐rich areas. GeoMx digital spatial profiling (DSP) revealed that regions colonized by *F. nucleatum* exhibit significant influence on the expression of RNAs and proteins involved in proliferation, migration and invasion. In vitro studies indicated that co‐culture with *F. nucleatum* significantly stimulates the proliferation and migration of breast cancer cells. Integrative spatial multi‐omics and co‐culture transcriptomic analyses highlighted the MAPK signalling pathways as key altered pathways. By intersecting these datasets, VEGFD and PAK1 emerged as critical upregulated proteins in *F. nucleatum*‐positive regions, showing strong positive correlations with MAPK pathway proteins. Moreover, the upregulation of VEGFD and PAK1 by *F. nucleatum* was confirmed in co‐culture experiments, and their knockdown significantly reduced *F. nucleatum*‐induced proliferation and migration. In conclusion, intratumoral microbiota in breast cancer exhibit significant spatial heterogeneity, with *F. nucleatum* colonization markedly altering tumour cell protein expression to promote progression and migration. These findings provide novel perspectives on the role of microbiota in breast cancer, identify potential therapeutic targets, and lay the foundation for future cancer treatments.

**Key points:**

- Intratumoral *Fusobacterium nucleatum* exhibits significant spatial heterogeneity within breast cancer tissues.
- *F. nucleatum* colonization alters the expression of key proteins involved in tumour progression and migration.
- The MAPK signalling pathway is a critical mediator of *F. nucleatum*‐induced breast cancer cell proliferation and migration.
- VEGFD and PAK1 are potential therapeutic targets to mitigate *F. nucleatum*‐induced tumour progression.

## Graphical abstract

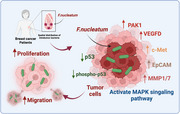

## 1 INTRODUCTION

The tumour microenvironment (TME) is a highly intricate system that consists of diverse cellular and structural elements, including tumour cells, immune cells, vasculature, and components of the extracellular matrix.^1^ These components collectively influence tumour growth, progression, and therapeutic response.^2^, ^3^ Recent research have identified the existence of microbiota within the TME, with certain micro‐organisms residing intracellularly.^4^, ^5^, ^6^, ^7^ In addition to genetic, epigenetic, and stromal factors, the host microbiota is essential in influencing cancer susceptibility and driving tumour progression.^8^, ^9^ In colorectal cancer, the intratumoral microbiota can influence colonic epithelial cells, cancer cells, and TME by inducing DNA damage, promoting apoptosis, and triggering epithelial‐to‐mesenchymal transition, which collectively contribute to cancer progression.^10^ Moreover, growing evidence indicates that micro‐organisms are present in various tumours previously considered sterile, including breast, pancreatic, and lung cancers.^4^, ^6^, ^11^, ^12^, ^13^, ^14^ For instance, in 2020, Nejman analyzed 1526 samples from tumour and adjacent normal tissue across seven different cancer types, revealing distinct intratumoral microbiota, which were predominantly found in tumour and immune cells^4^. Advancements in microbiota detection and analysis technologies have demonstrated that microbiota can colonize tumour tissues through mechanisms such as mucosal disruption, migration to adjacent tissues, and hematogenous invasion, thereby influencing tumour biological behaviour. Intratumoral microbiota can promote tumour initiation and progression via multiple mechanisms, including the induction of genomic instability and mutations, modulation of epigenetic changes, enhancement of inflammatory responses, evasion of immune surveillance, regulation of metabolic processes, and activation of signalling pathways that drive invasion and metastasis.^5^, ^6^, ^7^, ^12^, ^15^, ^16^, ^17^, ^18^, ^19^ These findings underscore the critical influence of the microbiota on tumour biology and its impact on clinical outcomes.

Breast cancer is the most prevalent cancer worldwide and continues to be the leading cause of cancer‐related deaths among women.^20^ It exhibits significant biological and clinical heterogeneity,^21^, ^22^ with potential risk factors including genetic susceptibility, lifestyle factors, exogenous hormone intake, endogenous hormone fluctuations, and the microbiota present within breast tissue.^23^, ^24^ Breast tumours harbor a more complex and varied bacterial microbiota than other tumour types.^4^ Bulk sequencing analysis revealed that breast cancer tissues possess distinct microbiota communities, characterized by higher abundances of *Porphyromonas*, *Lacibacter*, *Ezakiella*, *Fusobacterium*, and *Pseudomonas* compared to healthy controls, with these abundances further increasing in advanced‐stage tumours.^25^ In vitro and preclinical animal experiments indicate that tumour‐resident microbiota are emerging as critical components of breast cancer, exhibiting diverse biological functions that influence cancer progression, including DNA damage, chronic inflammation, and immune modulation.^26^, ^27^, ^28^ Recent research has demonstrated that bacteria present within the tumour can alter the actin cytoskeleton in circulating tumour cells, enhancing their ability to endure mechanical stress in the bloodstream and facilitating metastasis^17^. Additionally, *Fusobacterium nucleatum* promotes tumour progression and metastasis through the suppression of T cell infiltration.^16^ These findings highlight the critical impact of intratumoral microbiota in breast cancer susceptibility and progression.

Despite these advancements, most studies rely on bulk tissue analysis, hindering the precise identification of low‐biomass micro‐organisms in breast cancer. Due to their limited abundance, these micro‐organisms may exhibit spatial heterogeneity and execute a cancer‐promoting effect by acting specifically within the regions they inhabit. Therefore, investigating the spatial localization of microbiota and their interactions with host cells, which induce localized biological effects, is essential for accurately elucidating the pro‐carcinogenic roles and mechanisms of intratumoral micro‐organisms. In this research, we utilized a spatially resolved multi‐omics strategy to delineate the distribution of intratumoral microbiota within breast cancer tissues. Our findings revealed that localized *F. nucleatum* significantly alters protein expression patterns within the TME, particularly affecting MAPK signalling pathways. These alterations promote inflammatory responses and facilitate breast cancer progression and metastasis, providing new insights into microbiota‐TME interactions and identifying potential therapeutic targets for future interventions.

## 2 RESULTS

### 2.1 Spatial heterogeneity and distribution patterns of intratumoral microbiota in breast cancer

In this research, 5R 16S rRNA gene sequencing was conducted on 60 tumour tissue samples obtained from 15 patients diagnosed with invasive breast cancer (BC). This process generated 12 333 788 high‐quality sequences and identified 5757 operational taxonomic units (OTUs), with an average of 205,563 sequences and 96 OTUs per sample (Table S2). The rarefaction curves for various richness and diversity indices reached a plateau for all samples, confirming that the sequencing depth was adequate (Figure S2).

**FIGURE 1:**
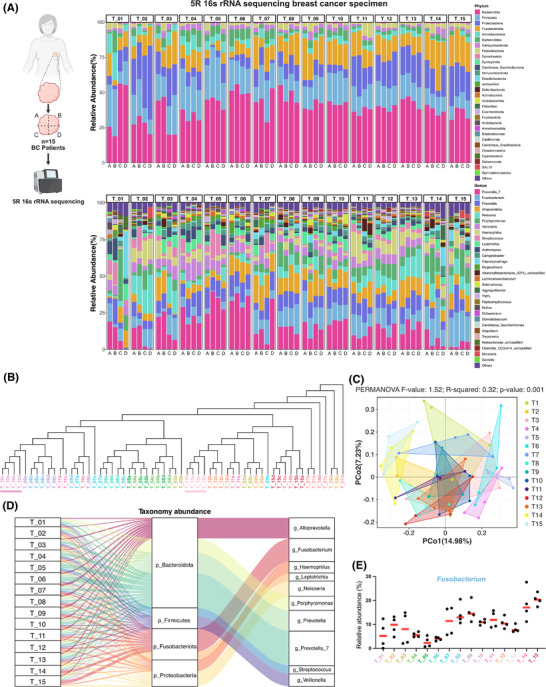
Heterogeneous distribution of intratumoral microbiota across tumour tissue. (A) Relative abundance of bacterial communities at the phylum and genus levels in each tumour tissue sample (*n* = 4 per patient) from 15 BC tumour specimens, identified by sequencing numerous 16S rRNA gene amplicons. Tumour tissues were divided into four parts (A–D) for each patient. The sequencing data were analyzed using QIIME2 (2024.05). (B) Dendrogram illustrating the clustering of genus‐level microbiome composition in tumour fragments. The dissimilarity index between samples was calculated using the Bray–Curtis test, and hierarchical clustering was performed using the Ward clustering algorithm to identify clustering of patient specimens (colour bars). Data were analyzed with R (version 4.1.3). (C) Principal component analysis (PCoA) plot representing the β‐diversity clustering of genus‐level bacterial communities from each piece of BC tumour tissue, along with permutational multivariate analysis of variance analysis (Bray–Curtis index). (D) Sankey plot showing the longitudinal composition of microbiota in tumour tissues from 15 BC patients. (E) Relative abundance of the *Fusobacterium* in each tumour mass from the 15 BC patients. Data are expressed as the mean and individual data points.

Dominant flora analysis (Figure 1A) identified 16 phyla in breast cancer tissues, with Bacteroidota (39.6%), Firmicutes (18.3%), Proteobacteria (14.3%), and Fusobacteriota (14.2%) being the most abundant. At the genus level, the most abundant species were *Prevotella* and *Fusobacterium*, which represented 15.6% and 10.5% of the total microbiota, respectively. Principal coordinate analysis (PCoA) of β‐diversity and clustering using the unweighted pair group method with arithmetic mean (UPGMA) (Figure 1B, C) demonstrated that the majority of patients (13 out of 15) displayed significant heterogeneity in the composition of their intratumoral microbiome, whereas only two patients showed a relatively stable intratumoral microbiome profile. This suggests that the spatial distribution of micro‐organisms within tumour tissue is largely heterogeneous. In order to identify bacterial taxa that may play key roles in the tumour microenvironment, we performed an in‐depth analysis of the dominant microbial communities.

The overall genus‐level distribution of the intratumoral microbiome across different patients' tissues is illustrated in the Sankey diagram (Figure 1D). Analysis of dominant genera across different regions of the same patient's tissues (Figure 1E) revealed significant variation in *Fusobacterium* abundance, indicating the heterogeneous spatial distribution of this dominant intratumoral microbiota within tumours. Notably, although spatially heterogeneous distributions were observed in top‐ranking genera such as *Prevotella_7* and *Alloprevotella*, our prioritization of *Fusobacterium* for in‐depth investigation was driven by three critical factors: First, its well‐defined species composition (notably *F. nucleatum*) provides a more tractable experimental model compared with taxonomically complex genera like *Prevotella_7* and *Alloprevotella*. Second, its remarkably high abundance (10.5% at the genus level) suggests potential ecological dominance within the tumour microenvironment. Third and most crucially, clinical epidemiological data reveal that *F. nucleatum* accounts for 53.8% of all *Fusobacterium*‐related infections,^29^ and extensive studies have demonstrated its mechanistic roles in multiple malignancies (e.g., colorectal cancer) through the modulation of tumour‐associated inflammation and immune evasion pathways. This robust clinical relevance establishes a strong rationale for investigating *F. nucleatum* in breast cancer pathogenesis, thus positioning it as the focal point of our subsequent research.

**FIGURE 2:**
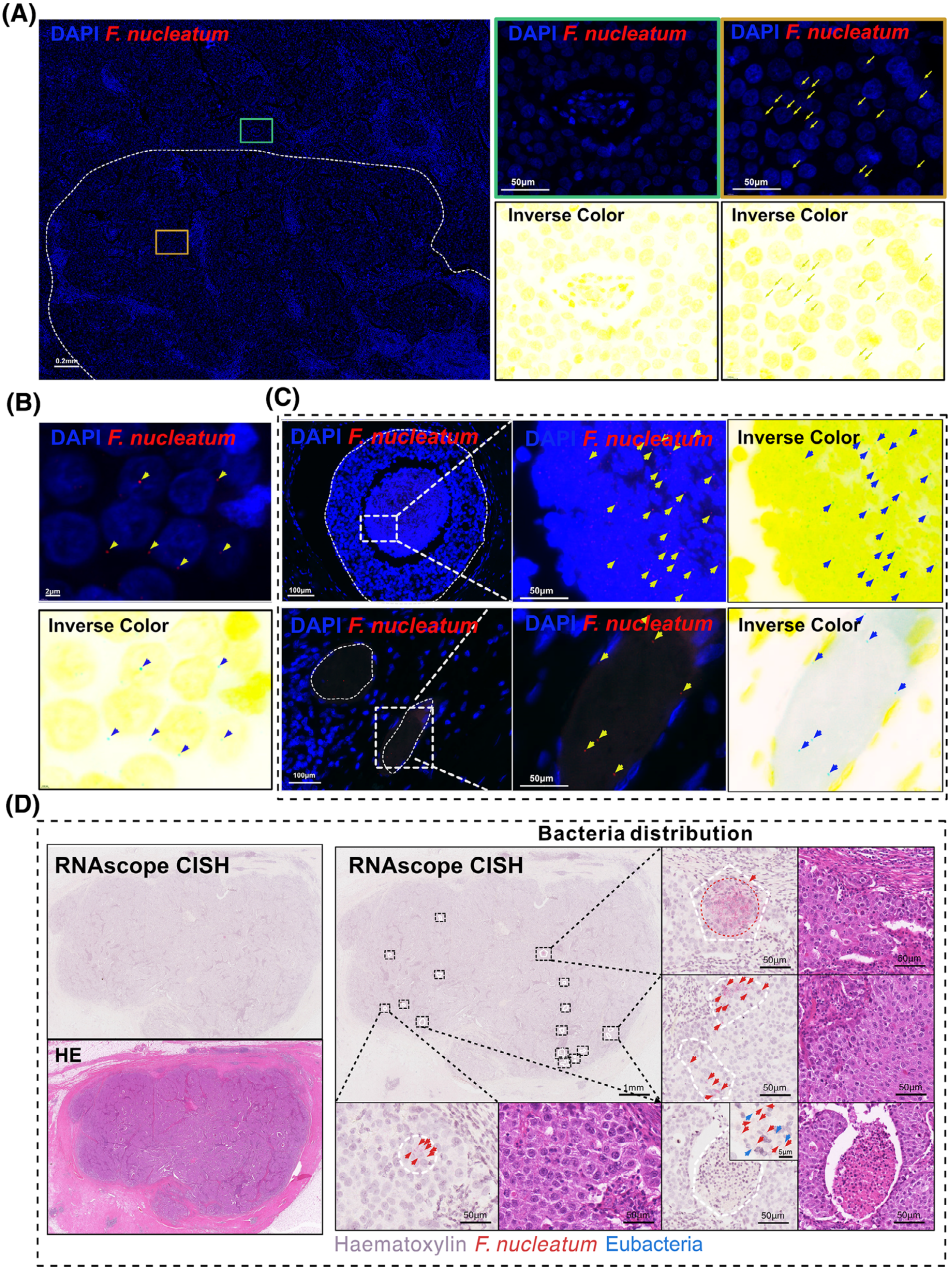
RNAscope FISH and CISH analyses demonstrate the heterogeneous distribution of *F. nucleatum* in breast tumour tissues. (A) RNAscope FISH analysis of tumour sections from BC patients. Inverse colour images are displayed to highlight bacteria vividly. Green box: *F. nucleatum*‐negative region. Yellow box: *F. nucleatum*‐positive region. Yellow arrows indicate *F. nucleatum* positivity. Red: *F. nucleatum* (ATCC25586) specific probe. Blue: DAPI. Scale bar: 200 µm (left), 50 µm (right). (B) RNAscope FISH analysis of BC tumour sections (as shown in A). Inverse colour images are displayed to highlight bacteria vividly. Yellow arrows indicate *F. nucleatum* positivity. Red: *F. nucleatum* (ATCC25586) specific probe. Blue: DAPI. Scale bar: 2 µm. (C) Upper left and right panels: RNAscope‐CISH images showing the spatial distribution of *F. nucleatum* in BC tumour tissue, with the *F. nucleatum* probe highlighted in red and the Eubacteria probe in green. Bottom left panel: Hematoxylin and eosin stain (H&E) of the RNAscope image. The scale bar is shown in the figure.

To further elucidate the spatial heterogeneity of *F. nucleatum*, we employed targeted RNAscope‐FISH/chromogenic in situ hybridization (CISH) staining to analyze tissues from five breast cancer patients. The selection of these samples was determined by the distinct distribution patterns of *Fusobacterium* observed in various tissue sections, as shown in Figure 1E. Among these, T1, T3, T7, T9, and T14 exhibited more pronounced differences in *Fusobacterium* distribution, and thus, these samples were selected for subsequent RNAscope‐FISH/CISH analysis. *F. nucleatum* exhibited significant regional distribution differences within the same field of view (Figure 2A), with the majority of punctate bacterial signals localized to the perinuclear areas of cells (Figure 2B). Additionally, visible light in situ hybridization imaging revealed a clear boundary between regions positive and negative for *F. nucleatum* (Figure 2D). These staining results partially confirmed the findings from the 5R 16S rRNA gene sequencing. Furthermore, *F. nucleatum* was partially enriched in necrotic cavities and vascular regions in two cases. This suggests a potential hematogenous dissemination route or niche adaptation to hypoxic necrotic microenvironments, though its prevalence and mechanisms require further validation (Figure 2C). Notably, the spatial distribution patterns of *F. nucleatum* within breast cancer tumours displayed marked heterogeneity, characterized by focal clustering in specific tumour regions.

### 2.2 Spatial multi‐omics profiling reveals distinct molecular pattern between *F. nucleatum*‐positive and *F. nucleatum*‐negative breast tumour regions

**FIGURE 3:**
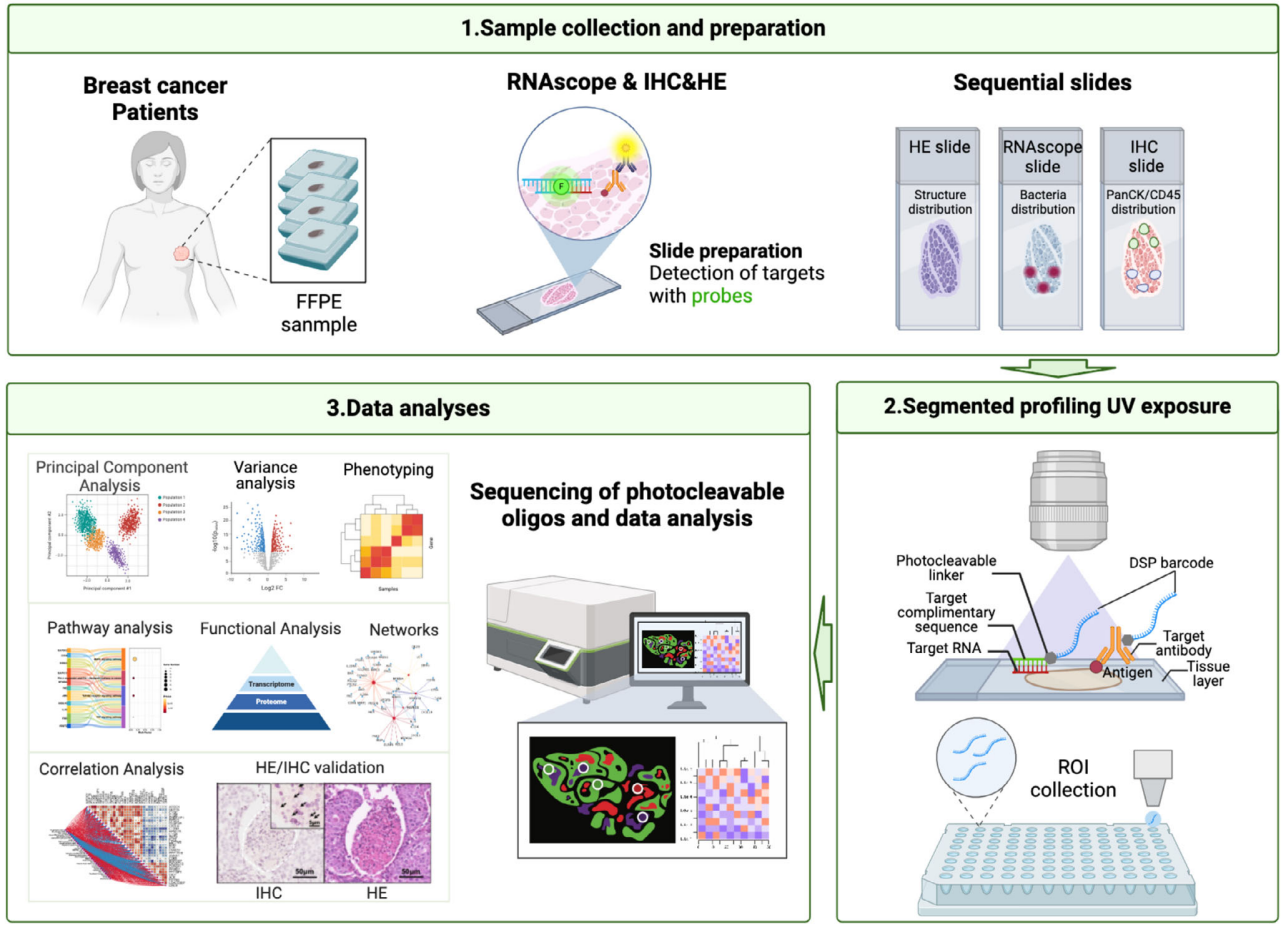
GeoMx digital spatial profiler (DSP) experimental protocols. GeoMx DSP was used to assess *F. nucleatum*‐related niches in BC tumours. 5 µm continuous FFPE sections were prepared from the tissue samples, three consecutive sections were selected for distinct analyses: one for H&E staining to assess tissue morphology, one for RNAscope CISH staining to identify the spatial distribution of *F. nucleatum*, and one for DSP analysis after the immunohistochemical (IHC) staining targeting immune (CD45+) and epithelial (PanCK+) compartments. Based on H&E and RNAscope CISH imaging of continuous tissue sections, regions of interest (ROIs) were delineated and categorized as either *F. nucleatum*‐positive or *F. nucleatum*‐negative. Samples were analyzed for *F. nucleatum*‐positive AOIs and *F. nucleatum*‐negative AOIs in the CD45+ and PanCK+ redions, involving the release of photocleavable barcode oligonucleotides for sequencing. The sequenced oligonucleotides provided spatial information about the respective RNA and protein targets in *F. nucleatum*‐positive or *F. nucleatum*‐negative regions.

Given the heterogeneous distribution of the intratumoral *F. nucleatum* within breast tumour tissues, our goal was to explore whether its spatial arrangement correlates with the heterogeneity observed in the tumour microenvironment (TME). To address this, we employed a spatial in situ multi‐omics approach, GeoMx DSP spatial multi‐omics, capable of efficiently profiling over 18 000 RNA transcripts and more than 570 proteins, to characterize the molecular patterns in *F. nucleatum*‐colonized versus noncolonized regions (Figure 3). Due to the high cost of GeoMx DSP technology and the observation in preliminary sequencing that T9 (Luminal B breast cancer) and T14 (triple‐negative breast cancer) exhibited distinct *Fusobacterium* distribution patterns and relatively high abundance, these two samples were selected for large‐scale tissue analysis. Specifically, to spatially resolve tumour cell‐dominant (PanCK+) and immune cell‐infiltrated (CD45+) regions, we performed fluorescence‐based tissue segmentation using PanCK‐ and CD45‐conjugated antibodies. Regions of interest (ROIs) were further classified as *F. nucleatum*‐positive or *F. nucleatum*‐negative through H&E and RNAscope CISH imaging of serial tissue sections.

**FIGURE 4:**
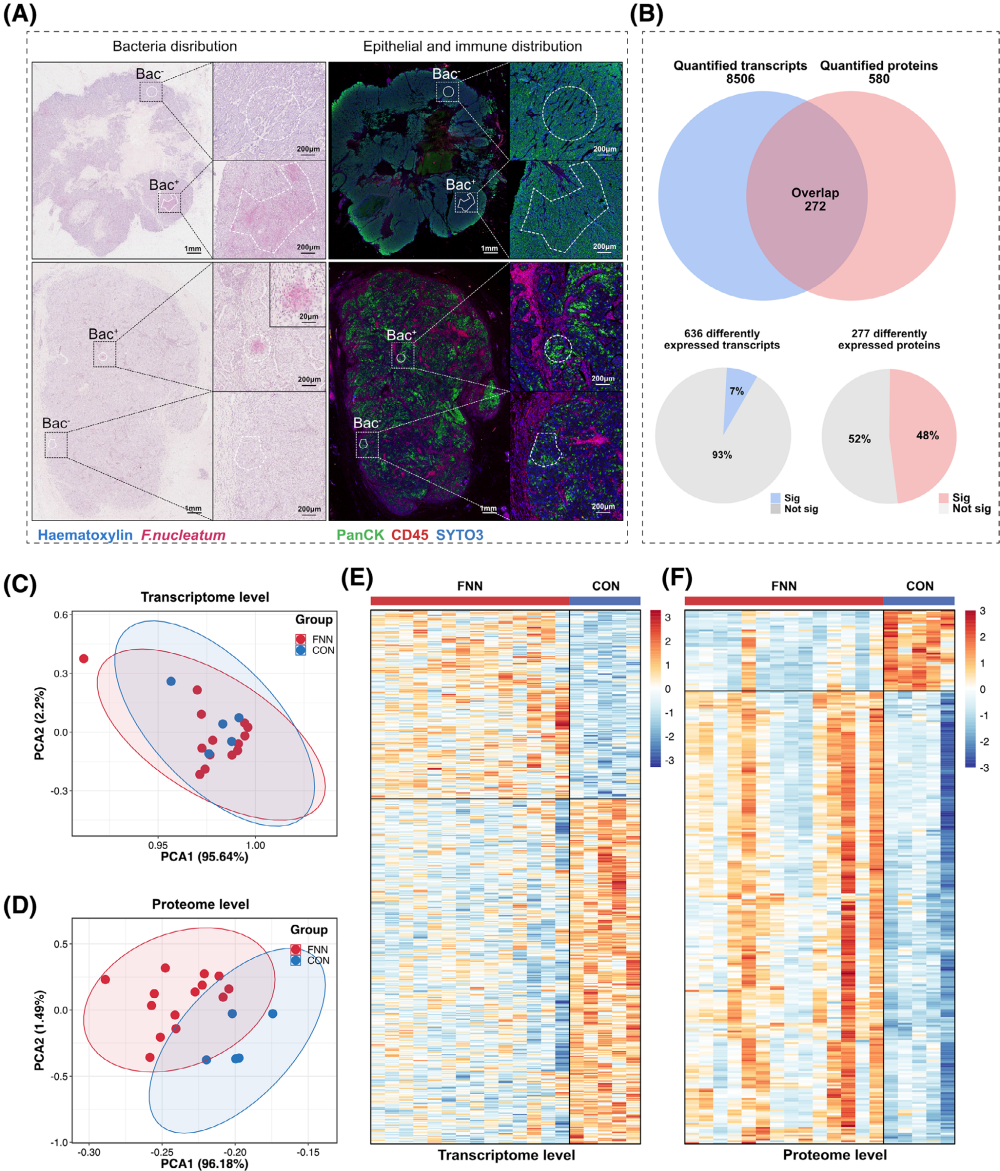
Spatial transcriptomic and proteomic analysis. (A) RNAscope‐CISH images showing the distribution of *F. nucleatum* (red) in BC tissue, with serial IHC images depicting the distribution of CD45+ (red) and PanCK+ (green) cells, identifying immune and epithelial compartments in BC tissue, respectively. The inset highlights representative AOIs for *F. nucleatum‐*positive and *F. nucleatum*‐negative areas with corresponding UV‐exposed regions. (B) Top: Gene overlap quantified at RNA and protein levels using OmicStudio tools (https://www.omicstudio.cn/tool). Bottom: Percentage of differentially expressed transcripts and proteins, calculated by significance (*p* < .05). (C, D) Principal component analysis of all quantified RNAs (C) and proteins (D). Data were analyzed using R (version 4.1.3). (E, F) Heatmaps showing differentially expressed transcripts (E) and proteins (F). Data were analyzed using R (version 4.1.3) and visualized with the ComplexHeatmap package.

Comparative analysis of immunohistochemistry (PanCK/CD45) and RNAscope CISH (*F. nucleatum*) revealed that *F. nucleatum* predominantly co‐localized with tumour cells (PanCK+) in 19 of 20 ROIs, whereas co‐localization with immune cells (CD45+) was observed in only 1 ROI (co‐localization rate: 95% vs. 5%). Given the pronounced spatial preference of *F. nucleatum* for tumour cells, subsequent mechanistic analyses focused on PanCK+/*F. nucleatum* co‐localized regions (Figure 4A). Additionally, in *F. nucleatum*‐positive ROIs, Eubacteria must be negative to ensure that the selected regions are specifically enriched for *F. nucleatum* without interference from other bacterial communities. As shown in Figure S4, other microbes were indeed observed in tumour tissues, but they were primarily distributed at a low density, interspersed with sparse *F. nucleatum*. Notably, we observed that *F. nucleatum* exhibited a distinct clustered distribution in its colonization regions, and these regions were almost devoid of colonization by other microbes. Ultimately, 19 ROIs (14 *F. nucleatum*‐positive ROIs and 5 *F. nucleatum*‐negative ROIs) were selected for transcriptomic and proteomic analysis.

**FIGURE 5:**
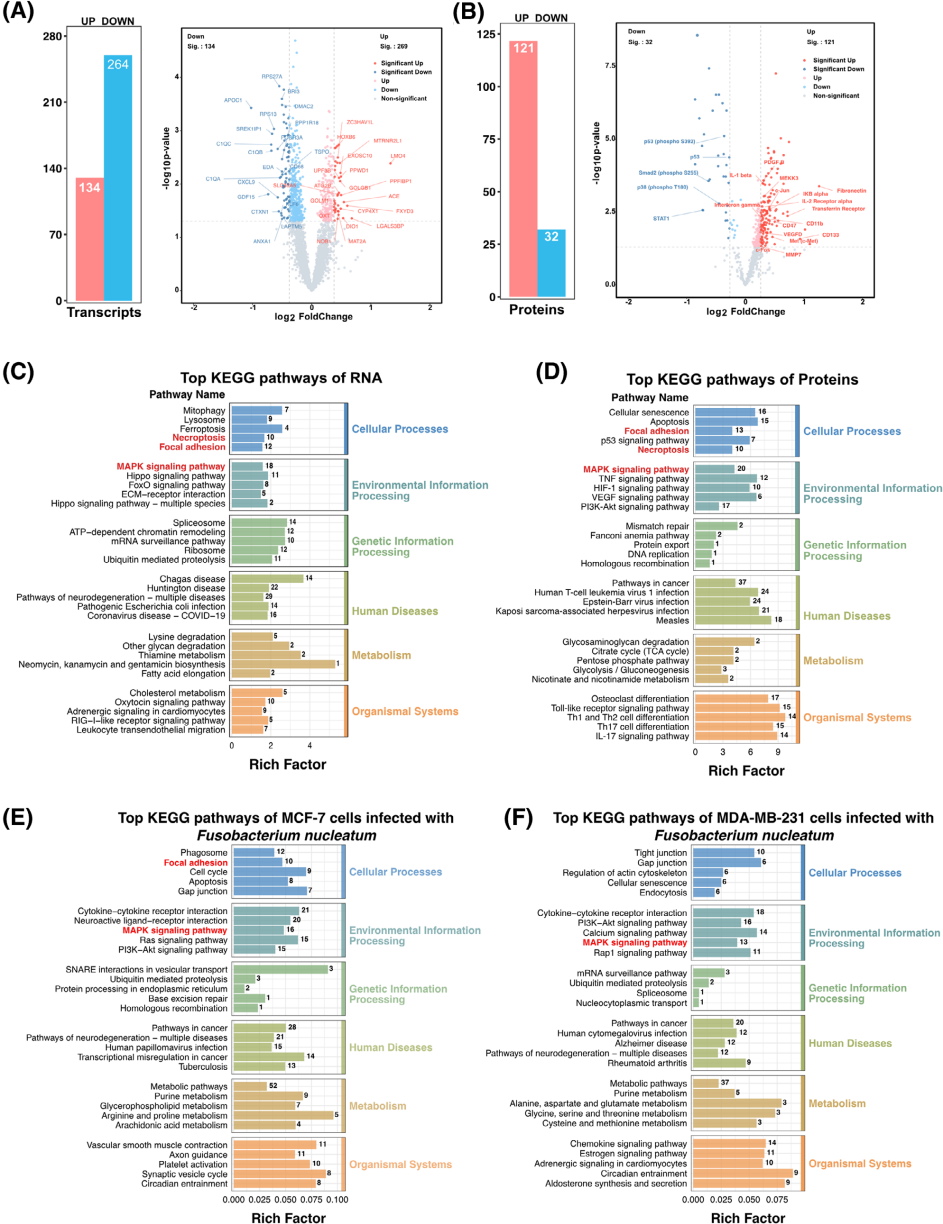
Differential and functional enrichment analysis of transcriptomics and proteomics. (A, B) Volcano plots showing differentially expressed transcripts (A) and proteins (B). Bar charts highlight significant changes in RNAs and proteins (*p* < .05, FC > 1.2). (C, D) KEGG pathway analysis of significantly different RNAs (C) and proteins (D). (E, F) KEGG pathway analysis of significantly different RNAs in MCF‐7 and MDA‐MB‐231 cells co‐cultured with *F. nucleatum*. Enrichment was performed using OmicStudio tools at https://www.omicstudio.cn/tool. Data were analyzed with R version 4.1.3 (ggplot2 package: 3.3.3).

Transcriptional and protein data were extracted from epithelial cancer cell regions within ROIs in the tissue, which were annotated as bacteria‐positive or bacteria‐negative using RNAscope‐CISH (Figure 4A). A total of 272 genes were consistently identified at both the transcriptomic and proteomic levels (Figure 4B). Principal component analysis (PCA) revealed no significant separation between the *F. nucleatum*‐positive (FNN) group and *F. nucleatum*‐negative (control) group at the transcriptomic level (Figure 4C), whereas a clear separation was evident at the proteomic level (Figure 4D). The lack of significant separation in PCA may be due to the subtle differences in the overall transcriptomic profiles across the groups, while the heatmap analysis revealed distinct alterations in gene and protein expression patterns that PCA could not capture. Both transcriptomic and proteomic heatmaps demonstrated notable variations in gene and protein expression between the FNN and control groups (Figure 4E, F). These findings imply that *F. nucleatum* may influence cellular processes through post‐transcriptional mechanisms and protein regulation. At the transcriptomic level, we identified 8606 RNAs (Table S3), of which 636 RNA levels were statistically significantly different (*p* < .05). Among these, 134 RNAs showed significantly higher expression, and 264 RNAs showed significantly lower expression (fold change >1.2 or <.83) in the FNN group (Figure 5A; Table S4). At the proteomic level, 574 proteins were identified (Table S5), with 277 proteins showing statistically significant differences (*p* < .05), including 129 proteins with significantly higher expression and 32 proteins significantly lower expression (fold change >1.2 or <.83) in FNN group (Figure 5B; Table S6). These results demonstrate that the presence of *F. nucleatum* leads to significant changes in both RNA and protein expression patterns, suggesting its potential involvement in influencing breast cancer cell biology.

As illustrated in the volcano plot, the PanCK+ region with *F. nucleatum* colonization exhibited significant upregulation of multiple pro‐tumour proteins and downregulation of several tumour‐suppressive proteins, collectively contributing to cancer progression through various molecular mechanisms (Figure 5B; Table S6). Tumour‐promoting proteins associated with cell proliferation and survival, such as active YAP1, Aurora A, CDKs (CDK1‐5), Cyclin A2, IGF2BP1/IMP1, KMT6/EZH2, c‐Met, PCNA, PDGFB, SIRT1, Syndecan‐1, transferrin receptor, and Oct4, were markedly elevated, enhancing cancer cell growth, inhibiting apoptosis, and accelerating cell cycle progression. Additionally, proteins that promote cell migration and invasion, including Cathepsin K, MMP1/7, Periostin, PAK1, Fibronectin, Integrin β1, Nectin‐4, and EpCAM, were upregulated, facilitating extracellular matrix degradation and increasing metastatic potential. Conversely, tumour‐suppressive proteins involved in DNA damage response and genomic stability (Figure 5B; Table S6), such as ATR (phospho S428), MSH2, and p53 (including phospho‐p53 S392), were significantly downregulated, leading to impaired DNA repair and increased genomic instability. Key cell cycle regulators, including phosphorylated Rb (S608, S807, T780) and CDK1, were decreased, potentially resulting in uncontrolled cell cycle progression. Furthermore, signal transduction proteins like STAT1 (phospho S727), STAT2, and Smad2 (phospho S255) were reduced, diminishing immune response activation and growth inhibition pathways. Histone modifications associated with gene repression, including acetylated Histone H2A/H3, methylated Histone H3, and phosphorylated Histone H3, were also decreased, indicating altered chromatin structure and gene expression regulation. Additionally, reduced expression of the glucocorticoid receptor (phospho S226), ROCK1/2, and Topoisomerase I suggests a decline in apoptotic signalling and an increase in cell survival mechanisms. Collectively, these alterations by *F. nucleatum* modulate key molecular pathways, promoting cancer cell proliferation, migration, and invasion, disrupting cell cycle control, and enhancing resistance to apoptosis, thereby contributing to the aggressive behaviour and progression of breast cancer.

### 2.3 Pathway and network analyses identify VEGFD and PAK1 as key mediators of *F. nucleatum*’s modulation of the tumour microenvironment

To elucidate the potential impact of *F. nucleatum* colonization on the local TME, we conducted a comprehensive analysis of the differentially expressed genes and proteins regulated by *F. nucleatum*. Using GO and KEGG enrichment analyses (Tables S7 and S8), we investigated the biological functions associated with these molecular changes. GO enrichment analysis of transcriptomic data revealed that the differentially expressed RNAs are involved in several key molecular functions and biological processes, including RNA binding, protein binding, peptidyl‐serine phosphorylation, biogenesis of ribosomal small subunits, and positive regulation of translation (Figure S6A). Similarly, the analysis of differentially expressed proteins identified significant molecular functions and biological processes such as protein binding, enzyme binding, positive regulation of gene expression, and the inflammatory response (Figure S6B). Furthermore, the differentially expressed genes and proteins localize to similar cellular components, including the nucleoplasm, cytosol, and extracellular exosomes.

To explore the signalling pathways potentially impacted, KEGG pathway enrichment analysis demonstrated that the genes and proteins with altered expression participate in multiple biological processes (Figure 5C, D). Importantly, these RNAs and proteins were notably concentrated in three major signalling pathways: MAPK signalling pathway (18 differential RNAs and 21 differential proteins), focal adhesion (12 RNAs and 13 proteins), and necroptosis (10 RNAs and 10 proteins) (Figure 5C, D). Additionally, transcriptomic profiling of the MDA‐MB‐231 (TNBC) and MCF‐7 (ER⁺) breast cancer cell lines, when co‐cultured with *F. nucleatum*, demonstrated a substantial enrichment of differentially expressed RNAs, particularly within the MAPK signalling pathway, in both cancer subtypes. Furthermore, in MCF‐7 cells, additional RNAs were enriched in the focal adhesion pathway (Figure 5E, F; Tables S9, S10, and S11). Collectively, these findings indicate that *F. nucleatum* influences the local TME through the regulation of multiple pathways, including MAPK and focal adhesion pathways, thereby exerting diverse regulatory effects on tumour progression.

**FIGURE 6:**
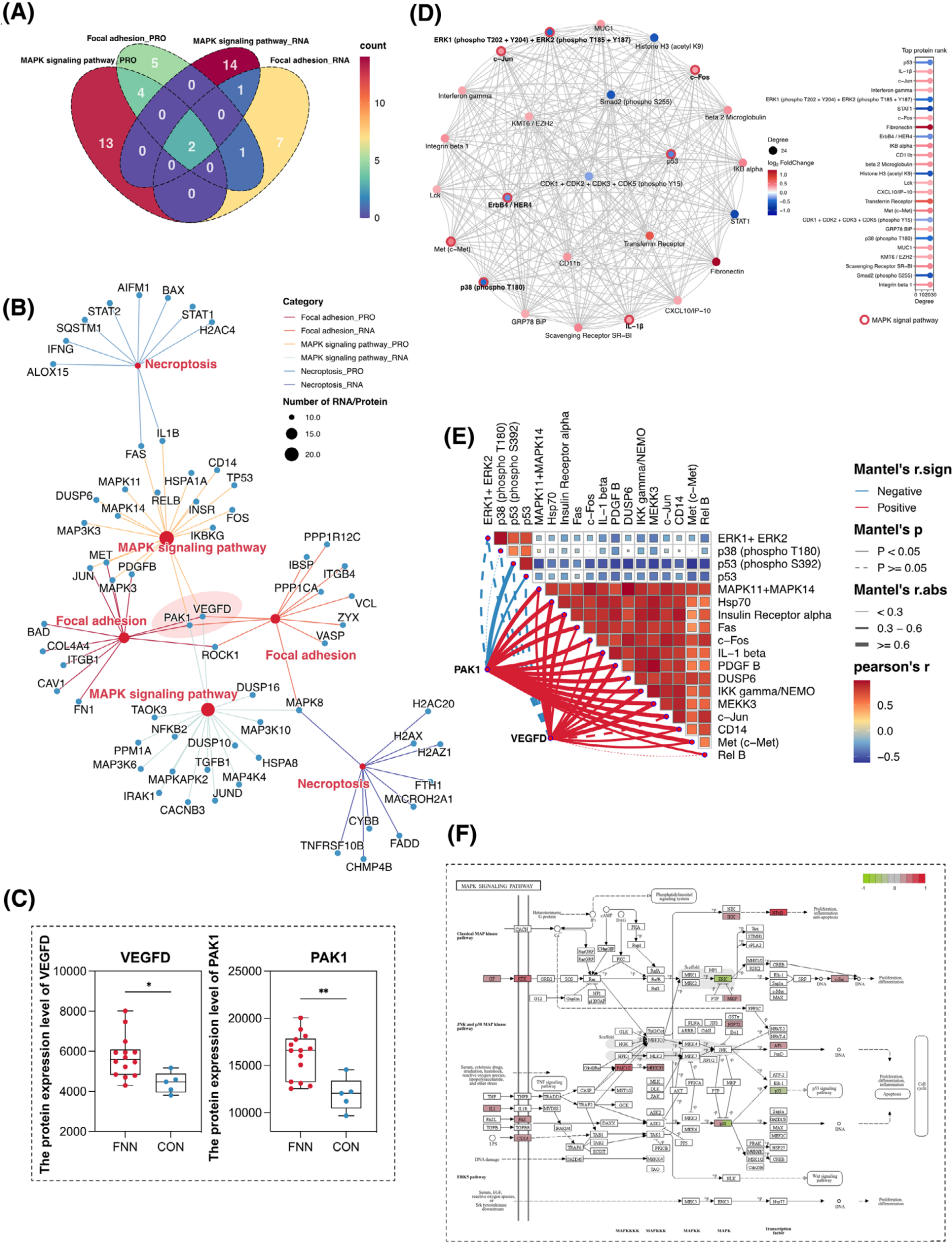
Key proteins and their role in signalling pathways. (A) Venn diagram showing intersecting genes of the MAPK and focal adhesion signalling pathways at the transcriptomic and proteomic levels. (B) Pathway network analysis of the MAPK signalling, focal adhesion, and necroptosis pathways at the transcriptomic and proteomic levels. (C) Differential expression of VEGFD and PAK1 in *F. nucleatum‐*positive and *F. nucleatum*‐negative tumour regions (*p < .05; ** *p* < .01). (D) Protein–protein interaction (PPI) network displaying associations among the top 20 ranked proteins. (E) Network heatmap showing the association of VEGFD and PAK1 with the top 20 important proteins. (F) Changes in significantly different proteins in the MAPK signalling pathway. Red indicates upregulation; green indicates downregulation. The statistical significance was calculated using the Student's *t*‐test.

To identify potential regulatory targets, Venn diagram analysis revealed that the MAPK and focal adhesion signalling pathways were co‐enriched by two identical RNAs/proteins at both the transcriptional and protein levels (Figure 6A). Pathway network analysis (Figure 6B) identified these two RNAs/proteins as VEGFD and PAK1, both of which were highly expressed in regions colonized by *F. nucleatum* (Figure 6C). To further investigate the role of differentially expressed proteins, we performed a protein–protein interaction (PPI) network analysis. The top 20 proteins based on their significance include MAPK11, MAPK14, c‐Fos, c‐Jun, PDGFB, and c‐Met (Figure 6D). Additionally, correlation heatmap analysis revealed a strong positive association between VEGFD, PAK1, and the top 20 proteins (predominantly key proteins in the MAPK signalling pathway) and significantly negatively correlated with P53 and phosphorylated P53 (Figure 6E). These findings indicate that VEGFD and PAK1 may serve as pivotal regulators linking *F. nucleatum* colonization to the activation of MAPK signalling pathways, thereby promoting breast cancer progression and metastasis. Furthermore, the MAPK signalling pathways are crucial for the pro‐tumour activities of *F. nucleatum* following colonization (Figure 6F).

### 2.4 *F. nucleatum* promotes proliferation and migration of breast cancer cells

**FIGURE 7:**
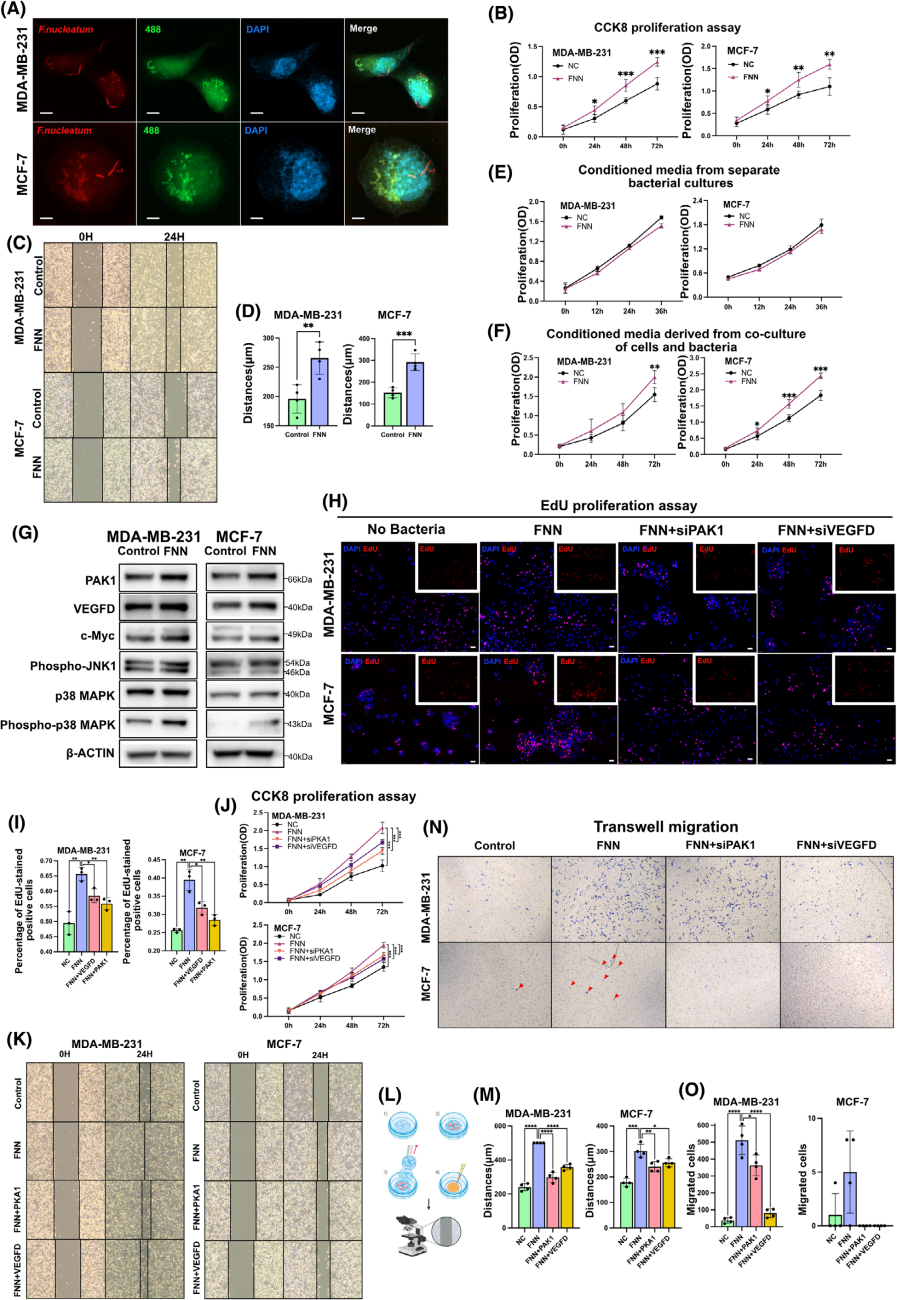
Mechanism of *F. nucleatum* promoting proliferation and migration of breast cancer cells. (A) Confocal microscopy showing spatial interaction between breast cancer cells (green: cytoskeleton; blue: nuclei) and *F. nucleatum* (red). Scale bar: 5 µm. (B) Line graph of CCK‐8 cell proliferation assay (*n* = 5; Student's *t*‐test). Statistical significance was determined by Student's *t*‐test (*p < .05; **p < .01; ***p < .001). (C) The wound‐healing assay was initiated with a uniform scratch width of .5 mm. (D) Migration distance quantification. Scratch width was measured at four predefined equidistant points per well at 0 and 24h using ImageJ, and distance was normalized to the initial width (0 h) (*n* = 4; Student's *t*‐test). (E, F) Line graph of CCK‐8 cell proliferation assay (*n* = 5; Student's *t*‐test). *p < .05; **p < .01; ***p < .001. (G) Western blot analysis of VEGFD, PAK1, and MAPK pathway proteins in MDA‐MB‐231 and MCF‐7 cells co‐cultured with *F. nucleatum*. Blots are representative of three biological replicates. (H, I) EdU proliferation assay: EdU staining (red: proliferating cells; blue: DAPI) (H) and quantification of EdU‐positive cells (I). Three independent replicates were analyzed (*n* = 3; Student's *t*‐test). (J) Line graph of CCK‐8 proliferation assay after siRNA interference (*n* = 5; Student's *t*‐test). *p < .05; **p < .01; ***p < .001. (K, M) Wound‐healing assay: Representative scratch images (K), schematic of the plate insert (L), and migration distance quantification (M). Scratch closure was measured at four equidistant positions per well (*n* = 4; Student's t‐test). *p < .05; **p < .01; ***p < .001. (N, O) Transwell migration assay: Crystal violet‐stained migrated cells (N) and quantification (O). Four independent experiments were performed (*n* = 4; Student's *t*‐test). *p < .05; **p < .01; ***p < .001.

Previous research has shown that *F. nucleatum* possesses strong invasiveness and is capable of surviving intracellularly, regardless of whether the host cells are epithelial, endothelial, keratin‐producing, or potentially immune cells.^30^, ^31^, ^32^, ^33^ In this research, we proved that *F. nucleatum* can adhere to and invade breast cancer cells. Laser confocal microscopy revealed *F. nucleatum* adhering to the surfaces of MCF‐7 and MDA‐MB‐231 (Figure 7A; Figure S7). Additionally, 3D laser confocal microscopy verified the presence of *F. nucleatum* within cells (Supporting Information Video).

To explore how *F. nucleatum* influences the biological behaviour of breast cancer cells, the MCF‐7 and MDA‐MB‐231 cell lines were co‐incubated with *F. nucleatum*. Cell proliferation was measured using the cell counting kit‐8 (CCK‐8) assay, and migration was evaluated by the wound healing assay. The results of the CCK‐8 assay demonstrated that co‐culture with *F. nucleatum* significantly enhanced the proliferation of MCF‐7 and MDA‐MB‐231 cells (*p* < .05) (Figure 7B). Moreover, the cell scratch assay demonstrated a marked increase in the migration capacity of these cell lines following co‐culture with *F. nucleatum* (*p* < .05) (Figure 7C, D). To verify the specificity of this effect, we conducted a similar co‐culture experiment with Escherichia coli (*E. coli*) as a control and found no significant effect on cell proliferation or migration (Figure S8). These findings indicate that the presence of *F. nucleatum* within tumours markedly influences the biological characteristics of tumour cells and potentially contributes to tumour progression and metastasis.

To determine whether the observed effects are directly caused by *F. nucleatum* or result from interactions following infection, we utilized supernatants from the *F. nucleatum* monoculture and from co‐cultures with MCF‐7 or MDA‐MB‐231 cells to stimulate breast cancer cell proliferation. The results of the CCK‐8 assay revealed that the supernatants derived from *F. nucleatum* monocultures alone had no significant impact on the proliferation of MCF‐7 and MDA‐MB‐231 cells. In contrast, the supernatants from co‐cultures with tumour cells significantly promoted their proliferation (Figure 7E, F). These results indicate that the colonization of *F. nucleatum* promotes breast cancer cell proliferation and migration by interacting with tumour cells, thereby potentially contributing to tumour progression and metastasis.

### 2.5 VEGFD and PAK1 play crucial roles in *F. nucleatum*‐promoted proliferation and migration of breast cancer cells

Based on GeoMx DSP spatial multi‐omics analysis, it was identified that VEGFD and PAK1 expression levels were markedly increased in breast cancer cells co‐cultured with *F. nucleatum*. Additionally, in the MDA‐MB‐231 and MCF‐7 breast cancer cell lines that were co‐cultured with *F. nucleatum*, key proteins involved in the MAPK signalling pathway, including c‐Myc, phospho‐JNK1, p38 MAPK, and phospho‐p38 MAPK, were markedly elevated, thereby confirming the results of the spatial multi‐omics analysis (Figure 7G). We hypothesized that VEGFD and PAK1 may play pivotal roles in the *F. nucleatum*‐promoted proliferation and migration of breast cancer cells. To validate this hypothesis, siRNA was used to suppress the expression of VEGFD and PAK1 in MCF‐7 and MDA‐MB‐231 cells (Figure S9). Both CCK‐8 and EdU assays demonstrated that the knockdown of VEGFD and PAK1 significantly decreased the proliferation stimulated by *F. nucleatum* in MCF‐7 and MDA‐MB‐231 cells (*p* < .05) (Figure 7H–J). Furthermore, transwell invasion and wound‐healing assays indicated that siRNA‐mediated suppression of VEGFD and PAK1 notably impaired the migratory and invasive behaviours of *F. nucleatum*‐activated MCF‐7 and MDA‐MB‐231 cells (*p* < .05) (Figure 7K–O). However, the proliferation and migration rates of these cells enhanced by *F. nucleatum* did not return to control levels following VEGFD and PAK1 knockdown. These results suggest that VEGFD and PAK1 are important mediators of *F. nucleatum*’s impact on breast cancer cell proliferation and migration.

## 3 DISCUSSION

Previously, tumour heterogeneity was mainly considered a result of intrinsic genetic alterations occurring in tumour cells during their processes of proliferation and expansion^34^. These genetic alterations have guided current primary cancer treatment strategies.^34^ However, since the 1990s, research has increasingly emphasized the role of extrinsic factors within TME, including a variety of non‐cancerous cells, such as fibroblasts, endothelial cells, and immune cells that infiltrate the tissue.^35^, ^36^, ^37^, ^38^ These cells interact with tumour cells, inducing transcriptional changes that contribute to tumour heterogeneity and regulate tumour progression and metastasis.^39^, ^40^, ^41^ As our understanding of the TME has deepened, so has knowledge of factors contributing to tumour heterogeneity. In addition to cellular components, diverse microbiota are present in the TME of most major tumour types.^4^, ^5^, ^6^, ^7^, ^19^, ^42^, ^43^, ^44^ These microbiota vary by tumour type, with specific bacteria influencing tumour progression and metastasis, thereby impacting therapeutic response and survival outcomes.^4^, ^5^, ^6^, ^18^, ^19^, ^45^, ^46^ However, the inherent heterogeneity of the TME complicates the analysis of interactions between its components, particularly between bacteria and host cells in tumour tissues. Advances in technologies such as RNA‐seq and GeoMx DSP spatial multi‐omics have facilitated the investigation of microbial–host interactions within the TME.^47^

In this study, 5R 16S rRNA sequencing revealed an uneven distribution of micro‐organisms within breast cancer tumours. This differential distribution suggests that intratumoral microbiota, as an essential element of the TME, may modulate the TME's biology in specific regions, influencing anti‐tumour immunity and tumour progression. Furthermore, sequencing analysis identified *F. nucleatum* as a major constituent of the breast cancer intratumoral microbiota. RNAscope FISH staining confirmed the focal, aggregated distribution of *F. nucleatum* in breast tumour tissues. *F. nucleatum* is a common oral bacterium closely linked to periodontal disease.^48^, ^49^, ^50^ Studies have demonstrated that *F. nucleatum* is abundant in multiple tumour types, including colorectal, pancreatic, oesophageal, and breast cancers, and its presence correlates with poorer survival outcomes in colorectal, pancreatic, and oesophageal cancers.^16^, ^51^, ^52^, ^53^, ^54^, ^55^, ^56^, ^57^, ^58^, ^59^ However, it is still uncertain whether the localized and clustered distribution of *F. nucleatum* within breast tumours impacts the surrounding TME, how this distribution alters the TME, and whether it plays a critical role in driving the progression and metastasis of breast cancer.

*F. nucleatum* is a Gram‐negative, anaerobic bacterium that adheres to surfaces and is predominantly located in the oral mucosa, where it is essential for biofilm formation.^60^ *F. nucleatum* can travel through the digestive tract and establish colonies in CRC tissues.^61^ Recent studies have also shown that *F. nucleatum* may colonize both colon and breast cancers via the bloodstream.^62^, ^63^ Using RNAscope FISH staining, we observed the presence of *F. nucleatum* in the blood vessels of breast cancer tissues, providing evidence of its colonization through the bloodstream. Additionally, *F. nucleatum* has been demonstrated to specifically interact with Gal‐GalNAc, a molecule that is expressed at levels 4.7× higher in breast cancer tissues compared with normal tissues.^16^ This may explain why *F. nucleatum* is among the most common micro‐organisms identified in breast tumours.

To further investigate the altered breast cancer TME due to the uneven distribution of *F. nucleatum*, we employed GeoMX DSP spatial multi‐omics analysis to compare *F. nucleatum‐*colonized sites with non‐colonized areas of breast tumours. In regions colonized by *F. nucleatum*, both spatial transcriptomes and proteomes were significantly enriched in three signalling pathways: MAPK signalling, focal adhesion, and necrotic apoptosis. Moreover, co‐culture experiments demonstrated significant enrichment of the MAPK signalling pathway in both MDA‐MB‐231 and MCF‐7 cells after co‐culture with *F. nucleatum*. The findings indicate that *F. nucleatum* may promote breast cancer progression through the activation of the MAPK signalling pathway. The MAPK signalling cascade plays a pivotal role in the regulation of cell proliferation and migration.^64^ MAPKs mediate communication between extracellular and intracellular signals, regulating key cellular processes.^65^ In mammals, the MAPK family is categorized into four main subgroups: ERK1/2, JNK1/2/3, p38 MAPKs, and ERK5.^65^ The p38 MAPK pathway is involved in regulating cell survival and enabling stress adaptation throughout tumour progression.^66^, ^67^, ^68^ MAPK14 serves dual essential functions in mammary carcinoma cells by ensuring genomic integrity and orchestrating DNA repair mechanisms.^69^, ^70^, ^71^ Additionally, MAPK11 facilitates the upregulation of lipid transport protein 2 (LCN2), a factor linked to increased tumour development, invasion, and metastasis, thereby contributing to cancer progression.^72^

Our proteomic analysis revealed increased expression of p38α (MAPK14) and p38β (MAPK11) in *F. nucleatum*‐colonized regions. This suggests that *F. nucleatum*‐enriched regions may enhance the growth and invasiveness of breast cancer cells when compared with areas lacking bacterial colonization. Furthermore, a notable reduction in the levels of p38 phospho‐T180 was observed in *F. nucleatum‐*colonized regions, and T180/Y182 phosphorylation of p38 has been proposed that they may serve a protective function in breast cancer.^73^, ^74^ These variations might stem from differences in p38MAPK expression patterns and substrate specificity, leading to functional diversity.

Key substrates in the p38MAPK pathway include transcription factors c‐Fos and c‐Jun, which are involved in regulating tumour progression^75^. Our findings revealed a significant upregulation of c‐Jun and c‐Fos expression in regions colonized by *F. nucleatum*. Importantly, these factors are key components of the AP‐1 complex,^76^ which regulates genes involved in processes such as tumour cell proliferation, apoptosis, and metastasis.^77^, ^78^, ^79^ Suwen Ou showed that infection with *F. nucleatum* enhances CRC cell migration by increasing MMP7 expression through the MAPK (JNK)‐AP1 pathway.^80^ Thus, we suggest that *F. nucleatum* could promote the growth and invasiveness of breast cancer cells by increasing the expression levels of c‐Jun and c‐Fos. Conversely, p53 expression was significantly reduced in *F. nucleatum‐*colonized regions. p53 is crucial for regulating processes like cell cycle progression, apoptosis, and genomic integrity. Therefore, decreased p53 expression in the *F. nucleatum* colonization regions may further enhance the malignant proliferative potential of tumour cells in these areas.^81^, ^82^

Based on transcriptomic and proteomic enrichment analyses, we found that VEGFD and PAK1 were significantly enriched at both transcriptional and protein levels and were highly expressed in regions colonized by *F. nucleatum*. VEGF‐D is a glycoprotein secreted by cells that initiates VEGFR‐2‐dependent signalling pathways, which are mainly involved in controlling the proliferation, migration and function of endothelial cells.^83^, ^84^ As reported by Achen et al., VEGF‐D, which is released by tumour cells, has the ability to activate VEGFR‐2 signalling in adjacent blood vessels.^85^ Furthermore, VEGF‐D may act not only through paracrine signalling but also via autocrine mechanisms, as observed in endometrial cancer^86^ and invasive cervical cancer,^87^ where its local production contributes to tumour progression, cell migration, and metastasis. *F. nucleatum* has been shown to modulate tumour cell migration by inducing neutrophil extracellular traps, which subsequently increase VEGF levels in CRC cells.^88^ In co‐culture experiments with breast cancer cells, we observed that *F. nucleatum* significantly promoted tumour cell proliferation and migration. Notably, VEGFD protein expression in tumour cells was significantly elevated, and VEGFD knockdown markedly downregulated the proliferation and migration, suggesting that VEGFD released by *F. nucleatum*‐colonized tumour cells is crucial for tumour proliferation and migration. However, this finding contradicts the conventional view that VEGF primarily affects tumour migration and invasion. VEGF may activate multiple signalling pathways in the complex tumour microenvironment, contributing to a proliferative phenotype. Bioinformatics analysis suggests that VEGF‐D triggers the activation of the MAPK pathway, thereby facilitating cell proliferation and enhancing cell survival.^89^ This aligns with our findings that increased VEGF secretion induced by *F. nucleatum* might be a key factor in activating the MAPK downstream pathway. Such activation could explain how VEGF indirectly affects cell proliferation and why *F. nucleatum* colonization significantly alters the local TME.

The PAK1 signalling pathway is essential for VEGF expression and its associated functions.^90^, ^91^ PAK1 is central to cell survival and the process of oncogenic transformation. Studies have indicated that amplification of the PAK1 gene occurs in around 30% of breast cancer cases.^92^, ^93^, ^94^ As an effector in the signalling pathways downstream of Cdc42 and Rac.^95^ PAK1 regulates cellular processes such as viability, adhesion, and movement.^96^ Through its kinase activity, PAK1 activates the RAF/MEK/MAPK pathway to promote proliferation,^97^ and its phosphorylation of MEK1 is key for focal adhesion formation, thereby enhancing tumour cell migration.^98^ Elevated PAK1 kinase activity correlates with an invasive phenotype in breast cancer cells.^96^ Our findings also indicated that PAK1 expression was upregulated in *F. nucleatum*‐colonized regions. Silencing PAK1 in *F. nucleatum*‐colonized breast cancer cells significantly reduced their proliferative and migratory capabilities. In line with our findings, numerous studies have shown that PAK1 overexpression enhances cell proliferation, motility, and invasiveness.99 Alterations in the protein expression patterns of tumour cells, including proteins such as VEGFD and PAK1, driven by *F. nucleatum* colonization, may activate key signalling pathways like MAPK, which are pivotal for the protumorigenic effects associated with *F. nucleatum* colonization.

## 4 CONCLUSION

In conclusion, although the overall microbiota abundance in tumours is minimal, their differential distribution and focal colonization as key components of the TME can substantially alter the local tumour milieu. *F. nucleatum*, a colonizing micro‐organism in breast tumours, significantly modifies the local microenvironment through its focal distribution. It influences the activation of downstream targets in the MAPK signalling pathway and focal adhesion by upregulating VEGFD and PAK1 expression. This, in turn, enhances the proliferation and migration of breast tumours. Investigating the effects of intratumoral *F. nucleatum* on the breast cancer microenvironment is essential for understanding the mechanisms underlying breast cancer progression and for developing future therapeutic strategies.

### 4.1 Limitations of the study

This study has several limitations. The sample size for the GeoMx DSP analysis was relatively small, which may affect the statistical significance and generalizability of the results. Additionally, *F. nucleatum* colonization was primarily observed in tumour cell regions, as well as in necrotic areas and blood vessels of the tumour based on a limited sample set, and interactions between *F. nucleatum* and immune cells were not examined. The origin of *F. nucleatum* and the factors driving its specific colonization patterns within the tumour remain unclear and require further investigation. Future studies will aim to include larger sample sizes and explore the detailed mechanisms of these signalling pathways more thoroughly, thereby enhancing the reliability and clinical relevance of the findings. Such follow‐up research will provide deeper insights into the role of microbiota in tumour development and could potentially identify novel therapeutic targets.

## 5 MATERIALS AND METHODS

### 5.1 Human sample collection

Tumor specimens were prospectively collected at Sir Run Run Shaw Hospital, Zhejiang University School of Medicine (Hangzhou, China), following the protocol approved by the Ethics Committee of the same institution (20220219‐30). Enrollment criteria for breast cancer patients included those aged 18–75 years, pathologically diagnosed with primary breast cancer, and had not received any treatment prior to tumour excision. Exclusion criteria included severe comorbidities, other active malignancies, known distant metastases, severe immunosuppression, pregnancy or lactation, and those considered medically or psychologically ineligible. Following informed consent procedures, 15 cases were enrolled with multi‐type specimen collection (including snap‐frozen and FFPE tissues) from each primary lesion, each assigned unique biospecimen identifiers (Table S1).

### 5.2 5R 16S sequencing and data analysis

Individual fresh‐frozen breast tumours (*n* = 15, Figure 1A) were divided into four equal portions. To ensure uniformity in tissue dissection, we employed a stereotactic division method. Fresh frozen tumours were placed in a sterile 3D grid mould (with 1 mm aperture spacing), and tissue sections were cut vertically along the sagittal and coronal planes to ensure equal volume for each piece (A–D). Each dissected tissue block (A–D) was stained with H&E and evaluated by a pathologist to confirm that only tumour parenchyma was present. Exclusion criteria included blocks containing dense lymphatic/vascular regions (CD31+/Podoplanin+), necrotic areas (>5% area), or calcified regions; transported in preservation boxes filled with dry ice, preserved in 1.5 mL tubes, and stored at −80°C. A novel approach was employed to perform 5R 16S rRNA amplification and sequencing that amplifies five regions of the 16S rRNA gene with multiplex amplification, providing higher resolution compared with traditional methods (Figure S1).^4^ Different types of negative controls were used, such as blank controls, DNA extraction controls, and controls without templates were processed following the same protocol as the tumour samples to monitor bacterial contamination introduced during sample collection and processing. The sequencing of the libraries was carried out using the Illumina NovaSeq 6000 platform. Reads from individual samples were demultiplexed, quality‐filtered, and mapped to the five amplified regions according to their primer sequences. The SMURF (Short Multidimensional Sequencing Region Framework) methodology was implemented to integrate sequencing data across five targeted zones within a consolidated analytical framework, addressing complex statistical challenges through maximum likelihood optimization.^100^ Taxonomic classification was conducted using the GreenGenes reference database (released May 2013). Comprehensive bioinformatics processing involved the MicrobiomeAnalyst platform for microbial profile visualization, β‐diversity evaluations implemented through Bray–Curtis dissimilarity metrics, dimensionality reduction via PCA, permutational multivariate analysis of variance, and hierarchical clustering dendrograms using Ward's linkage method.^101^

### 5.3 RNAscope FISH

The spatial profiling of *F. nucleatum* within neoplastic tissues was accomplished using the RNAscope 2.5HD multiplex fluorescence detection system (Advanced Cell Diagnostics). The standardized protocol encompassed four critical phases: (1) tissue processing with a Leica RM2255 automated microtome (Leica Microsystems) to generate 5 µm FFPE sections mounted on electrostatic adhesion slides (Superfrost Plus) 16 h ambient desiccation; (2) pre‐hybridization conditioning involving 60 min thermal equilibration at 60°C in a HybEZ II automated hybridization station (Advanced Cell Diagnostics), followed by three‐stage solvent treatment (xylene/anhydrous ethanol) and endogenous enzymatic blockade with 3% H_2_O_2_ (10 min RT incubation). Antigen retrieval required optimization complete immersion in 200 mL RNAscope 1X Target Retrieval solution within an Oster steam heating apparatus, maintaining thermal at exposure nearing conditions (99 ± 1°C) for 15 min. Post‐retrieval processing comprised sequential dehydration in absolute ethanol (3 min), ambient duration air‐drying, and establishment of hydrophobic demarcation around tumour regions barrier using pens. Finally, the slides were treated with RNAscope Protease Plus at 40°C for 20 min in a HybEZ II hybridization oven. Each slide was treated with 150 µL of RNAscope probe mixture targeting *F. nucleatum* (B‐Fusobacterium‐23S‐3zz‐C2; Advanced Cell Diagnostics). For negative and positive controls, corresponding serial slides were incubated with probes targeting unrelated genes (item numbers: 321641 and 320751). The probe hybridization protocol was executed in a thermal‐regulated hybridization chamber (HybEZ II Automated System, Advanced Cell Diagnostics) under controlled parameters: 40°C incubation for a 120 min hybridization duration. Post three‐stage signal amplification, *F. nucleatum*‐C2‐specific labelling was achieved through Opal 570 fluorophore application (200 µL volume) with 30 min thermal incubation at identical temperature. Intermittent processing included dual 2 min rinses in 1X RNAscope wash buffer following each amplification/colouration cycle. Terminal procedures involved (1) applying DAPI nuclear counterstain (ThermoFisher Scientific) for 30 s ambient staining and (2) slide preservation using ProLong Gold Antifade Mountant (ThermoFisher Scientific). Digital imaging was acquired via 3DHISTECH Pannoramic MIDI II whole‐slide scanner (Hungary) equipped with 40× objective, employing spectral unmatching protocols: Opal 570 excitation at 550 nm wavelength (illumination duration 3.96 ms) and DAPI detection at 370 nm (3.83 ms exposure).

### 5.4 RNAscope CISH

To precisely demarcate regions for GeoMx spatial profiling, architectural visualization was achieved through chromogenic detection using the RNAscope 2.5 HD multiplex system (Advanced Cell Diagnostics), enabling dual assessment of histological features and *F. nucleatum* colonization patterns. The standardized preparation protocol involved three sequential phases: (1) sectioning FFPE blocks into 5 µm slices with a Leica RM2255 automated microtome (Leica Microsystems); (2) mounting sections on charged adhesion slides (Superfrost Plus) for 16 h ambient dehydration; (3) performing thermal stabilization at 60°C for 60 min in a HybEZ II automated hybridization chamber (Advanced Cell Diagnostics). Deparaffinization, blocking of endogenous peroxidase, and antigen retrieval were carried out as previously described (refer to RNAscope FISH). Upon completion of preparatory procedures, the mounted sections underwent targeted proteolytic conditioning in a HybEZ II automated hybridization station (Advanced Cell Diagnostics). The optimized protocol specified: application of RNAscope protease‐enriched solution under precisely controlled thermal parameters (40 ± .5°C) for a standardized 20 min incubation interval, then 150 µL of a probe mixture was added, including a probe targeting *F. nucleatum* (Fusobacterium‐23S‐3zz‐C2, Advanced Cell Diagnostics) and a Eubacteria probe targeting the conserved 16S‐rRNA region (EB‐16S‐rRNA‐C1, Advanced Cell Diagnostics). Probe hybridization was performed in a HybEZ II hybridization chamber at 40°C for 2 h. After six amplification steps, *F. nucleatum*‐C2 channels were visualized using the red detection reagent. Slides were rinsed twice with 1X RNAscope Wash Buffer for 2 min per wash between each amplification and visualization step. Subsequently, after an additional four hybridization steps, the Eubacteria‐C1 signal was visualized using the green detection reagent. To validate assay specificity and sensitivity, consecutive tissue sections underwent parallel processing with RNAscope 2‐plex Negative Control Probes (cat# 320751; Advanced Cell Diagnostics) and species‐matched Positive Control Probes (cat# 321641). Nuclear counterstaining was performed by immersing slides in 50% Gill's hematoxylin solution for 30 s, followed by 30 s of deionized water rinsing under continuous flow. The slides were dried to remove excess water and sealed with resin. Images were acquired by scanning RNAscope CISH slides with a 3DHISTECH Pannoramic MIDI II using a 40× objective.

### 5.5 GeoMx digital spatial profiling

This employed the study GeoMx Digital Spatial Profiler (DSP) to analyze formalin‐fixed paraffin‐embedded (FFPE) specimens obtained from 15 breast cancer (BC) cases The experimental protocol comprised three critical phases: (1) target localization guided by RNAscope CISH imaging to demarcate *F. nucleatum*‐positive and *F. nucleatum*‐negative areas of interest (AOIs) within neoplastic tissues; (2) immunofluorescence co‐staining with pan‐cytokeratin (CKPan) and CD45 antibodies for‐ tumourimmune cell demarcation across serial sections. Spatial multi‐omics profiling integrated the GeoMx Human Whole Transcriptome Atlas with Immune Oncology Proteome Atlas modules, enabling concurrent quantification of >18 000 RNA species and 570 protein targets within predefined AOIs. All hybridization probes and immunoreagents UV carried sensitive oligonucleotide barcodes. Technical specifications included 5 µm FFPE section preparation involving deparaffinization, 20 min thermal antigen retrieval at 100°C, and 15 min protein digestion with 1 µg/ml Proteinase K (Advanced Cell Diagnostics) at 37°C. Overnight probe hybridization at 4 nM concentration was followed by two 25 min washes with 50% formamide/2×SSC buffer (37°C) and five sequential 1‐min rinses in ×1TBS‐T. Post‐hybridization procedures involved tissue sealing with NanoString's Buffer W during 1 h horizontal incubation at ambient temperature. Detection required the preparation of an antibody cocktail containing morphology markers in Buffer W, with each immunoreagent uniquely barcoded. Post‐blocking removal, the detection mixture was administered onto slides for 16 h cold incubation (4°C) under humidified conditions. Subsequent processing encompassed: triple 10 min TBS‐T washes, 30 min room temperature fixation with 4% paraformaldehyde, dual 5 min TBS‐T rinses, and nuclear counterstaining using 500 nM SYTO13 for 15 min at ambient temperature prior to GeoMx platform loading. Then, the slides were scanned in the GeoMx platform. Circular ROIs with a diameter of 500 µm were selected for each tissue section, and a pathologist annotated each ROI to estimate the percentage of tumour and stromal content. The spatial distance between ROIs ranged from 164 to 36 252 µm, with a median of 15 981 µm (Figure S5A). High‐throughput sequencing of genetic barcodes in each ROI revealed a median of 12 986 678 successfully matched probe reads per ROI, with an interquartile range of 9 644 355 to 23 333 811 (Figure S5B). Analysis of the normalized gene expression density in each ROI indicated homogeneity without significant deviation, ensuring data reliability and stability (Figure S5C,D). The segmentation analysis option was used to obtain oligonucleotides separately from the immune and epithelial regions. The cut oligonucleotides were collected by microcapillary aspiration using the GeoMx Digital Spatial Profiler v2.1 software and stored at −20°C for sequencing.

### 5.6 Library preparation and sequencing

For PCR amplification of oligonucleotides from each ROI, a forward primer (CAAGCAGAAGACGGCATACGAGATXXXXXXXXXXGTGACTGGAGTTCAGACGTGTGCTCTTCCGATCT) and a reverse primer (AATGATACGGCGACCACCGAGATCTACACXXXXXXXXXXACACTCTTTCCCTACACGACGCTCTCTTCCGATCT) were utilized, where “X” denotes unique double‐indexed Illumina i5/i7 sequences customized to retain the identity of each ROI. Amplified PCR products were pooled and subjected to dual‐stage purification with AMPure XP magnetic particles (Beckman Coulter). RNA‐derived and protein‐associated amplicons were individually barcoded to construct discrete sequencing libraries. Quantitative analysis of library integrity and molarity was performed on a 2100 Bioanalyzer system (Agilent Technologies). High‐throughput paired‐end sequencing (2 × 150 bp) was executed using the NextSeq 550 system (Illumina) following standardized protocols.

### 5.7 GeoMx DSP data processing and analysis

Post‐sequencing raw data underwent preprocessing steps, including adapter trimming, read merging, and sequence alignment for probe identification. Molecular barcode (UMI) tracking technology effectively removed amplification artefacts and redundant sequences, converting filtered data into digital quantification metrics. Gene‐level quantification was achieved by aggregating probe measurements per biological specimen, with final values determined as median signals after outlier probe exclusion. The limit of quantification was established as the logarithmic average of negative control probe signals plus twice the standard deviation. Pathway enrichment analysis employed the standardized scoring algorithm in the GSVA computational framework (v1.32.0), utilizing background‐corrected log2‐transformed expression values as input. Inter‐sample dissimilarity was quantified through L2 norm‐based distance metric. Antibody specificity assessment defined the signal‐to‐noise ratio (SNR) as target signal intensity relative to the mean intensity of triplicate IgG‐negative controls within matching regions, with SNR <3 indicating undetectable targets. Spatial proteomic data normalization incorporated three reference proteins (ribosomal S6, histone H3, and glyceraldehyde‐3‐phosphate dehydrogenase) for signal calibration. Differential gene expression profiles between bacterial‐positive and bacterial‐negative AOIs were analyzed using the GeoMx Data Analysis software (NanoString; version 2.5.0.145). Fold changes and *p*‐values were reported for each gene. Statistical analyses conducted in R were performed in version 3.6.2 with RStudio version 2022.02.3+492.

### 5.8 Cell line culture conditions

Two human‐derived breast carcinoma cell models (MDA‐MB‐231 and MCF‐7) were procured from the Cell Bank of Chinese Academy of Sciences (Shanghai branch). Standard culture protocols involved Dulbecco's MEM (Gibco, USA) enriched with 10% FBS (fetal bovine serum, same supplier) and 1% antibiotic‐antimycotic cocktail. For bacterial co‐culture investigations, the experimental system utilized McCoy's 5A medium (Gibco), supplemented with an equivalent serum concentration. Cellular maintenance was conducted under controlled environmental parameters (37°C, 5% CO₂, humidified atmosphere), with subculture intervals (48–72 h) determined by confluency monitoring.

### 5.9 Bacterial strains and culture conditions

The experimental procedures employed *F. nucleatum* subsp. nucleatum strain ATCC 25586 (Fnn) for cellular investigations. Bacterial cultivation was conducted on 5% Columbia blood agar base (Oxoid) supplemented with defibrinated ovine erythrocytes (Novamed), maintained in an anaerobic workstation (Bactron I‐II, Shellab) under a controlled atmosphere (90% N₂, 5% CO₂, 5% H₂) at 37°C for 48 h incubation. Species verification was performed through MALDI‐TOF mass spectrometry (Bruker microflex LT/SH) on isolated colonies. Selected clones were subsequently inoculated into CBHK broth for 24 h anaerobic cultivation at 37°C. The bacterial growth phase was monitored spectrophotometrically at OD600, with target values adjusted between .6 and .8 to ensure exponential phase synchronization. A pre‐calibrated standard curve established through optical density measurements enabled precise bacterial quantification. All experimental protocols maintained a 50:1 bacterium‐to‐host cell ratio as predetermined infection parameters.

### 5.10 Cell transcriptome sequencing and data analysis

Human breast cancer cell lines MCF‐7 and MDA‐MB‐231 were co‐cultured with *F. nucleatum* following the established protocol. Experimental groups with matched controls were designed in quintuplicate biological replicates. After 24 h co‐culture, cellular samples were collected for transcriptomic profiling. Total RNA isolation was performed using TRIzol reagent (Thermo Fisher, 15596018) following standard protocols. RNA quantification and quality assessment (NanoDrop ND‐1000, Thermo Scientific, Wilmington, DE) for concentration (A260/A280 1.8–2.1) and purity evaluation, with RNA integrity verification (RIN > 8.0) using a bioanalyzer system (2100 Bioanalyzer, Agilent). Polyadenylated mRNA was enriched through dual‐round oligo(dT) magnetic bead selection (Thermo Fisher, 61011), followed by fragmentation via metal ion hydrolysis. First‐strand cDNA synthesis employed SuperScript II reverse transcriptase (Invitrogen, 18064014), with subsequent double‐stranded cDNA generation using Klenow fragment. Size‐selected fragments (200–400 bp) were processed with UDG enzyme (NEB, M0280S) for strand‐specific library construction. High‐throughput sequencing was performed on the NovaSeq 6000 system (Illumina) in 150 bp paired‐end configuration by LC‐Bio. Primary sequencing outputs underwent quality filtration (removing adapter sequences and low‐quality reads), followed by reference genome alignment (GRCh38.p13) using Hisat2. Differential gene expression analysis was conducted with DESeq2, while functional enrichment analysis (GO/KEGG) utilized the clusterProfiler computational pipeline.

### 5.11 Confocal scanning microscopy

This investigation employed laser scanning confocal microscopy to analyze co‐culture systems of *F. nucleatum* with human breast cancer cell lines (MCF‐7 and MDA‐MB‐231). Following 24 h co‐incubation at a defined infection ratio (multiplicity of infection [MOI] = 50:1), the cellular specimens underwent triple‐rinsing with phosphate‐buffered saline (PBS) prior to 30 min fixation using 4% paraformaldehyde under ambient conditions. Post‐fixation procedures included three PBS washing cycles and subsequent membrane permeabilization with .2% Triton X‐100 for 4 min at room temperature. The permeabilized specimens were then subjected to nuclear and cytoskeletal staining through 20 min incubation with dual fluorescent probes targeting nuclear DNA (NucBlue ReadyProbes, Invitrogen) and actin filaments (ActinGreen 488, same manufacturer). Concurrently, bacterial membrane structures in suspension were labelled with FM 4–64 (FXMolecular Probes) at 5 µg/mL. After completing the staining protocol with triple PBS washes, specimens were treated with 10 µL anti‐fade mounting medium. Three‐dimensional architectural reconstruction of infected cells was achieved through z‐stack imaging using the Leica SP8 confocal microscope system (Leica).

### 5.12 siRNAs transfection

VEGFD‐targeted and PAK1‐specific siRNAs, incorporating a scrambled control siRNA, were commercially produced through custom synthesis services provided by RiboBio Co., Ltd. Transfections were performed using the RiboBio transfection reagent (RiboBio), following the protocol provided by the manufacturer. Forty‐eight hours post‐transfection, total cellular proteins were extracted to evaluate the efficiency of the transfection.

### 5.13 Protein extraction and western blotting

Confluent adherent cells were subjected to triple washing with ice‐chilled PBS prior to lysis with RIPA buffer (Beyotime Biotechnology, cat# P0013B) containing protease inhibitor and phosphatase inhibitors under 4°C conditions, following standardized extraction protocols. Protein quantification was performed via bicinchoninic acid assay (Thermo Scientific, Cat# 23225) using a microplate reader (BioTek Synergy H1). Western blotting procedures were optimized based on established methodology^102^: equal protein aliquots (20 µg/lane) were electrophoresed on 10% SDS‐PAGE gels and subsequently electroblotted onto PVDF membranes (Millipore, IPVH00010) using wet transfer conditions (200 mA, 90 min). Sequential immunoblotting included incubation with primary antibodies targeting VEGFD (MCE, HY‐P82640, 1:500), PAK1 (MCE, HY‐P81618, 1:500), c‐Myc (CST, 5606S, 1:1000), phospho‐p38 MAPK (CST, 4511S, 1:1000), total p38 MAPK (CST, 9212S, 1:1000), phospho‐JNK1 (abcam, ab215208, 1:1000), with ACTIN (CST, #4970L, 1:1000) serving as loading control. HRP‐conjugated anti‐rabbit IgG secondary antibody (CST, 7074S) was applied at 1:5000 dilution. Chemiluminescent signals were captured using an imaging system (Bio‐Rad ChemiDoc MP) and analyzed with Image Lab 6.1 software.

### 5.14 Cell viability assay

The breast cancer cell lines MDA‐MB‐231 and MCF‐7 were employed in this study. Cells were seeded in 96‐well culture plates at 5 × 10^3^ cells/well (triplicate wells per condition) with 12 h pre‐culturing for monolayer formation. Bacterial co‐culture was initiated by exposing cells to *F. nucleatum* and *E. coli* suspensions at a multiplicity of infection (MOI) of 50:1 for 4 h. Post‐infection cellular viability was evaluated at 24, 48, and 72 h intervals. Proliferation kinetics were determined using the CCK‐8 assay (Dojindo Molecular Technologies; cat# CK04) following manufacturer guidelines. Absorbance measurements at 450 nm were conducted using a full‐spectrum microplate reader (Multiskan GO, Thermo Fisher Scientific).

EdU incorporation assays utilized four‐well chamber slides (Lab‐Tek II System, Thermo Scientific). Following 12 h adhesion, cells were infected by *F. nucleatum* (MOI = 50:1) for 24 h co‐culture. The 2X EdU working solution (final 10 µM) was prepared by diluting the stock solution (10 mM) 1:1000 in a complete medium. A pre‐equilibrated working solution was dispensed at equal volume to achieve the final concentration, followed by 2 h pulse labelling. Post‐incubation, cellular fixation was performed with 4% paraformaldehyde (15 min, RT) and permeabilized through three 3 min rinsing cycles with PBS‐T (0.1% Tween‐20). Membrane permeabilization employed 0.3% Triton X‐100 (10 min, RT) with subsequent washing replicates. Nuclear counterstaining used 1× Hoechst 33342 solution (10 min, dark conditions) prior to confocal microscopy imaging. Next, 1 mL 1X Hoechst 33342 solution was added to each well in the dark at RT for 10 min. Wash with 1 mL washing solution per well for 3 min. Fluorescence was then detected using a Leica SP8 fluorescence microscope, and EdU‐positive cells were counted using Fiji 1.0 software.

### 5.15 Cell migration assays

Transwell migration analysis was performed utilizing permeable cell culture chambers (Corning Inc.). The experimental protocol consisted of the following steps: Harvested cells in the logarithmic growth phase were trypsinized and resuspended in FBS‐free culture medium. A 300 µL aliquot containing 3 × 10⁵ cells/mL were dispensed into the apical compartment, while 600 µL of the serum‐deprived medium was introduced into the basolateral chamber. Following cellular attachment, the apical medium was replaced with an FBS‐free solution containing *F. nucleatum* and *E. coli* at a multiplicity of infection (MOI = 50:1) for 4 h co‐incubation. Post‐co‐culture, both compartments were aspirated and replenished with 300 µL serum‐free medium in the apical side and 600 µL chemoattractant medium containing 12% FBS in the basolateral chamber. After 24 h incubation under standard conditions (37°C, 5% CO₂), chambers underwent PBS washing twice per compartment. Non‐migratory cells located on the upper membrane surface were mechanically removed through swabbing with sterile cotton applicators. Subsequently, the migrated cells remaining attached to the lower membrane surface underwent fixation in 4% paraformaldehyde followed by 5 min staining with 0.1% crystal violet solution. Following triple PBS rinsing cycles, cell migration was documented via microscopic imaging and quantitatively analyzed using Fiji 1.0 image processing software.

### 5.16 Cell scratch assay

The cellular suspension underwent gradient centrifugation (150×*g* for 5 min) to remove cellular debris and non‐viable cells. Following quantification with a cell counter, the suspension was standardized to a density of 3 × 10^5^ cells per millilitre, with 110 µL aliquots dispensed into four‐compartment culture inserts (ibidi GmbH). In the post‐attachment phase, cultures underwent medium replacement with a fresh solution containing *F. nucleatum* at MOI = 50:1. Following 4 h of co‐culture, the culture existing medium from each well was aspirated and substituted with fresh medium devoid of *F. nucleatum*. Subsequently, cellular specimens underwent 24 h incubation under controlled conditions (37°C, 5% CO_2_). Once the cells had properly attached, the cell scratch insert was gently removed using sterilized tweezers. Then, each 35 mm culture dish received 2 mL of serum‐depleted medium renewal, initiating a subsequent 24 h culture cycle. Systematic observations using an inverted phase contrast microscope were conducted at the preset time points, with image acquisition simultaneously digitized for subsequent analysis.

### 5.17 Statistical analyses

The data analysis was performed with SPSS Statistics (Version 26.0; IBM, released 2019) and R programming environment (Version 3.5.2; R Foundation for Statistical Computing). Gene expression variations were analyzed through the Mann–Whitney *U* test, Student's *t*‐test, or Welch's adjusted *t*‐test, selected based on data characteristics. All cellular experiments were conducted in three independent biological replicates, with between‐group differences calculated via two‐sample *t*‐test. For multiple comparison corrections, the raw *p*‐values underwent false discovery rate adjustment following the Benjamini–Hochberg procedure. Statistical significance thresholds for two‐tailed tests were established as: ns (*p* > .05), * (*p* ≤ .05), ** (*p* ≤ .01), *** (*p* ≤ .001), and **** (*p* ≤ .0001).

## AUTHOR CONTRIBUTIONS

**Jun Zhang and Xinyou Xie**: Conceptualization, project administration, supervision, funding acquisition, and writing—review and editing. **Feng Zhao and Rui An**: Methodology, sample collection, validation, data curation, formal analysis, software, writing—original draft, and writing—review and editing. **Haitao Yu**: Methodology and data curation. **Yilei Ma and Shaobo Yu**: Methodology and writing—reviewing and editing. **Haitao Yu and Yanzhong Wang**: Resources. **Yuzhen Gao**: Methodology. The authors read and approved the final manuscript.

## CONFLICT OF INTEREST STATEMENT

The authors declare no conflict of interest.

## Supporting information



Supporting Information

Supporting Information

Supporting Information

Supporting Information

## Data Availability

All sequencing data generated and analyzed during this study are publicly available in the Sequence Read Archive (SRA) under the accession numbers PRJNA1157313 and PRJNA1163343. Additional data supporting the findings of this study are available from the corresponding author upon reasonable request.
