# Supplementary material for: Integrated spatial multi‐omics profiling of Fusobacterium nucleatum in breast cancer unveils its role in tumour microenvironment modulation and cancer progression
Source: Clin Transl Med. 2025 Mar 11;15(3):e70273. doi: 10.1002/ctm2.70273 (PMC11897063; doi:10.1002/ctm2.70273)
Supplement: Supplementary file 1 — Supporting Information [file CTM2-15-e70273-s001.docx]

Supporting Information

Impacts of Intratumoral Spatial Heterogeneity of *Fusobacterium nucleatum* on Breast Cancer Progression

Feng Zhao#, Rui An#, Yilei Ma, Yuzhen Gao, Shaobo Yu, Yanzhong Wang, Xinyou Xie* and Jun Zhang*

**Supplementaryary Figure S1: 16S rRNA sequencing method:** The 5R 16S sequencing approach was employed, involving multiplex PCR amplification and sequencing of five regions on the 16S rRNA gene. Compared to the conventional v3-v4 region amplification strategy, this method amplifies regions that cover about 68% of the full-length 16S sequence, significantly improving the coverage and resolution of bacterial species detection, especially for microbial samples with low biomass.

**Supplementary Figure S2: Relative bacterial richness and evenness analyses.** **A.** The Chao1 index estimating the number of OTUs contained in the sample. **B.** Rarefaction curve evaluating the relative bacterial richness to determine whether further sequencing would identify additional OTUs. **C.** The Shannon index estimating the microbial diversity index in the samples. **D.** The Simpson index estimating the index of microbial diversity in the sample. **E.** Good coverage represents the coverage of each sample library, and the higher the value, the higher the probability that the sequence is detected in the sample. **F.** Pielou's evenness index (Pielou_e) is a measure of the evenness of species distribution in a community. It reflects how the abundance of different species varies.

**Supplementary Figure S3: RNAscope FISH reveals that Fusobacterium nucleatum is distributed both in the necrotic areas of the tumor and within the tumor vasculature.** **A.** The necrotic areas of the tumor exhibit abundant Fusobacterium nucleatum positivity. Red: Fusobacterium nucleatum, blue: DAPI. **B.** Fusobacterium nucleatum is detected in the vascularized regions of the tumor. Red: Fusobacterium nucleatum, blue: DAPI.

**Supplementary Figure S4:** RNAscope-CISH images displaying the spatial distribution of bacteria in BC tumor tissue. The red signal highlights *F. nucleatum*, while the blue signal marks Eubacteria (universal bacterial probe)

**Supplementary Figure S5: GeoMx DSP spatial multi-omics experiments: A.** Spatial distance between any two ROIs in tumor tissue (Unit: μm). **B.** Median number of successfully matched probe reads per ROI. **C.** Analysis of normalized gene expression density distribution across each ROI showed homogeneity in gene expression, with no significant deviation.

**Supplementary Figure S6: GO enrichment analyses of significantly different RNAs and proteins.** A. Left: GO enrichment analyses of significantly different RNAs. Right: GO enrichment analyses of significantly different proteins.

**Supplementary Figure S7: Confocal microscopy images.** Red: Fusobacterium nucleatum, green: cytoskeleton, blue: DAPI.

**Supplementary Figure S8: Effects of *E. coli* infection on the proliferation, migration, and MAPK signaling activation in breast cancer cell lines MDA-MB-231 and MCF-7. A-B.** CCK-8 proliferation assay. Data represent mean ± SD of three independent experiments (n=5). Statistical significance was determined by Student’s t-test (*P < 0.05; **P < 0.01; ***P < 0.001). **C-D.** Transwell migration assay: Crystal violet-stained migrated cells (C) and quantification (D). Four independent experiments were performed (n=4; Student’s t-test). **E.** Western blot analysis of P38 MAPK, Phosphor-JNK, and β-Actin proteins in MDA-MB-231 and MCF-7 cells co-cultured with *E. coli*. Blots are representative of three biological replicates. **F.** The densitometry data of the WB analysis

**Supplementary Figure S9: Western blotting (WB) analysis showing the expression levels of VEGFD and PAK1 proteins following siRNA interference.** **Left panel:** MDA-MB-231 cell line subjected to siRNA interference targeting PAK1, with three different siRNAs tested. The siRNA with the most effective interference, siPAK1-3 and siVEGFD-2, was selected for further analysis based on densitometry analysis. **Right panel:** MCF-7 cell line subjected to siRNA interference targeting PAK1, with three different siRNAs tested. The siRNA with the most effective interference, siPAK1-1 and siVEGFD-3, was selected for further analysis based on densitometry analysis.


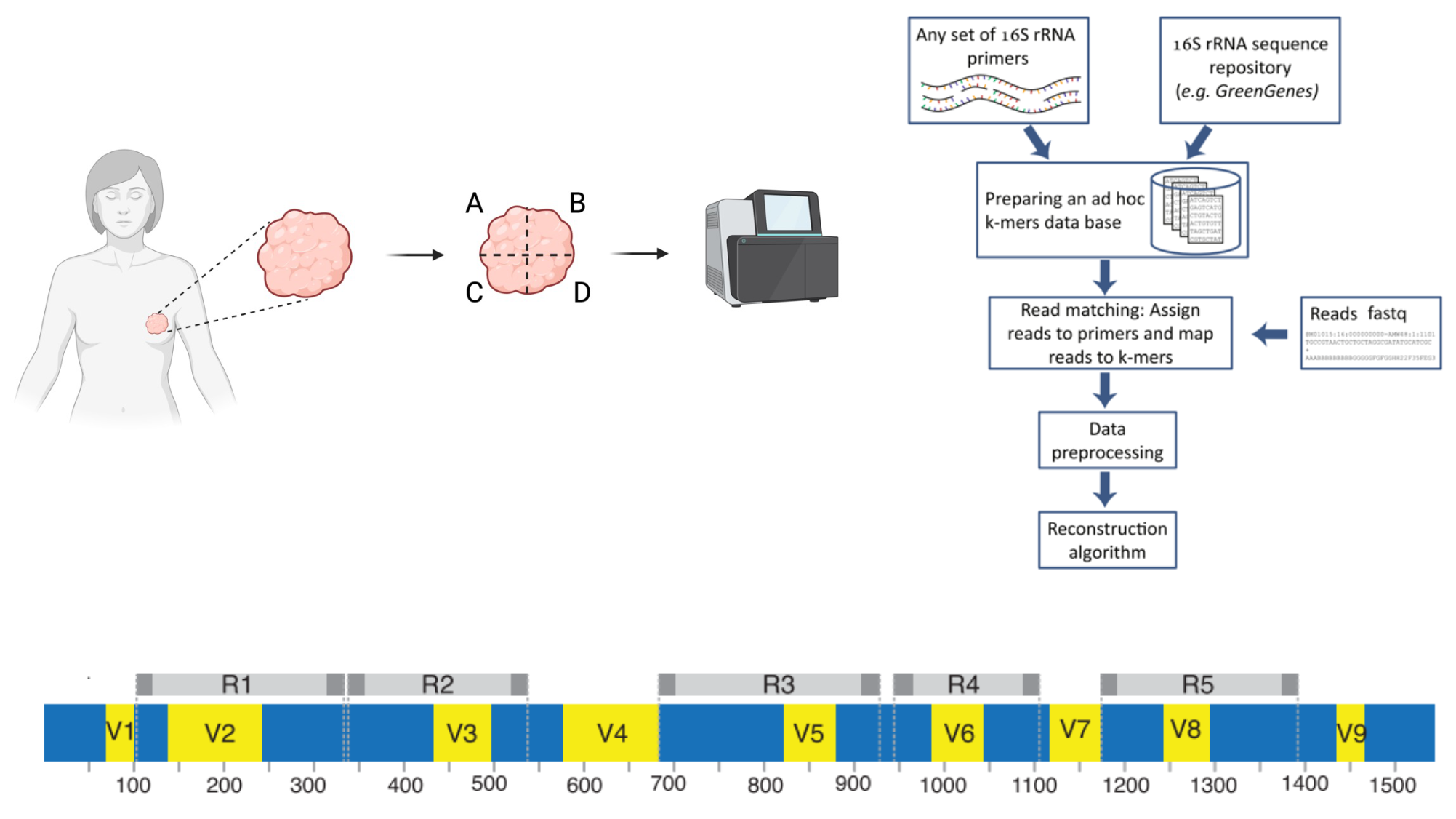


**Supplementary Figure S1: 16S rRNA sequencing method**


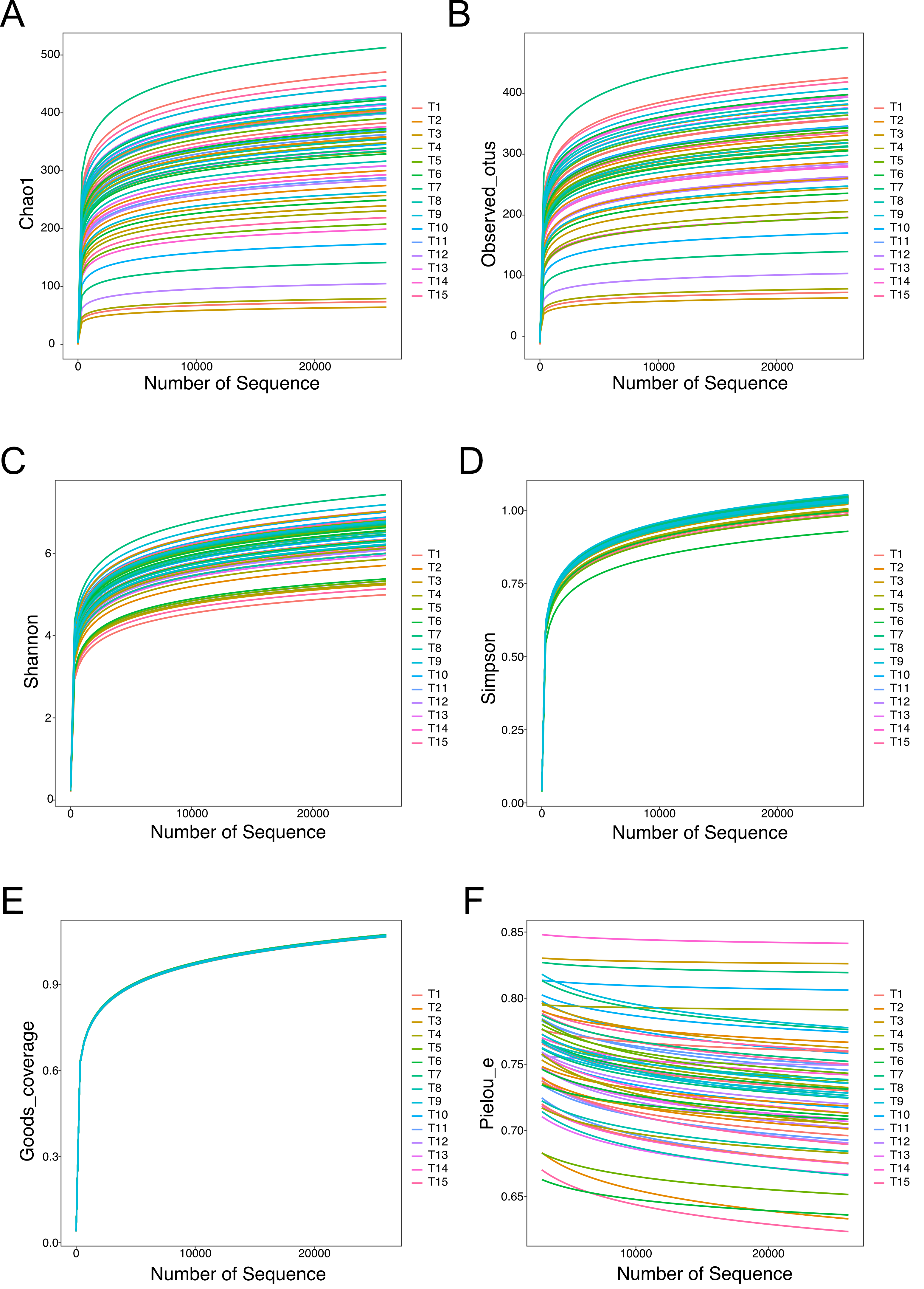


**Supplementary Figure S2: Relative bacterial richness and evenness analyses.**


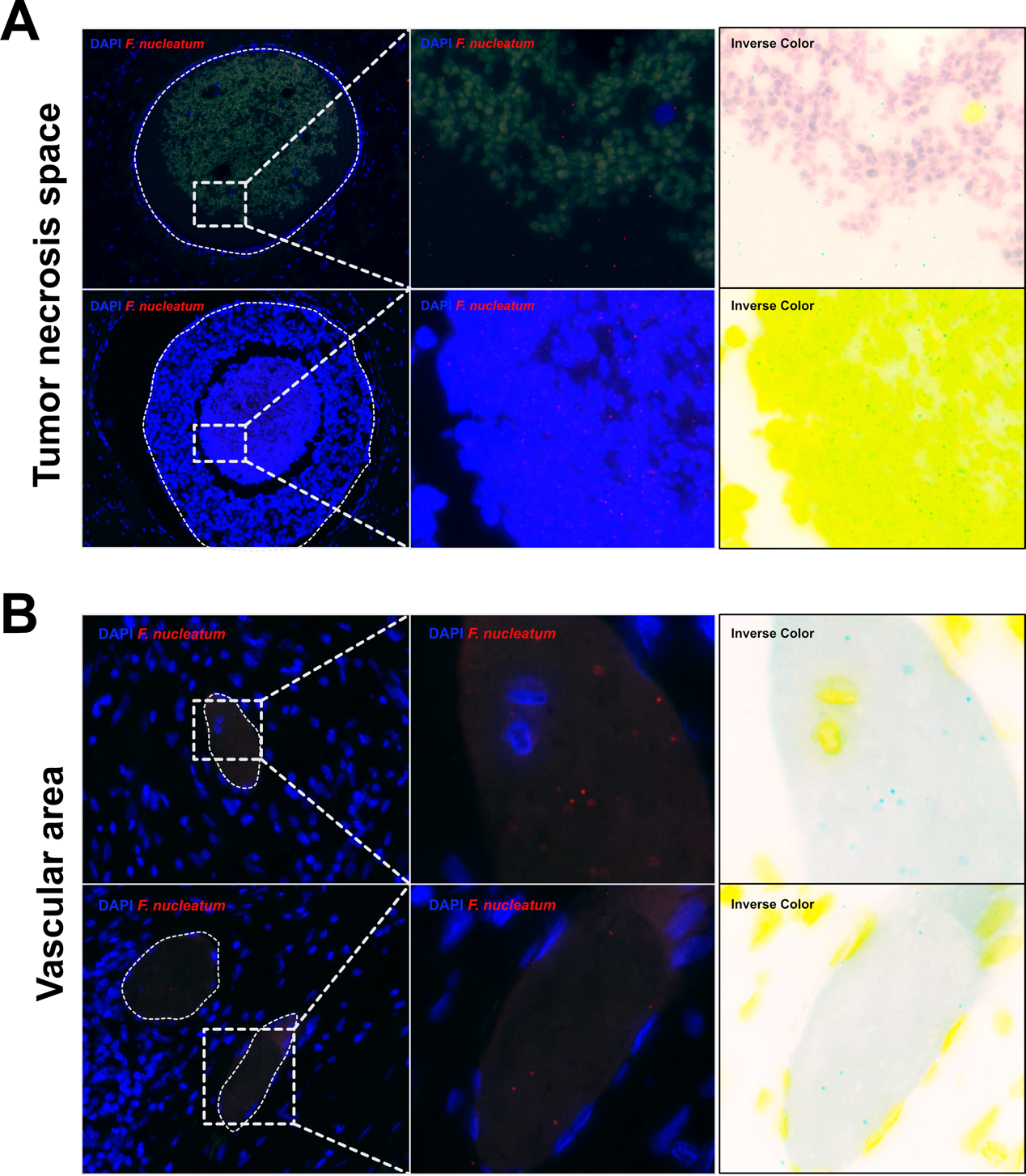


**Supplementary Figure S3: RNAscope FISH reveals that Fusobacterium nucleatum is distributed both in the necrotic areas of the tumor and within the tumor vasculature.**


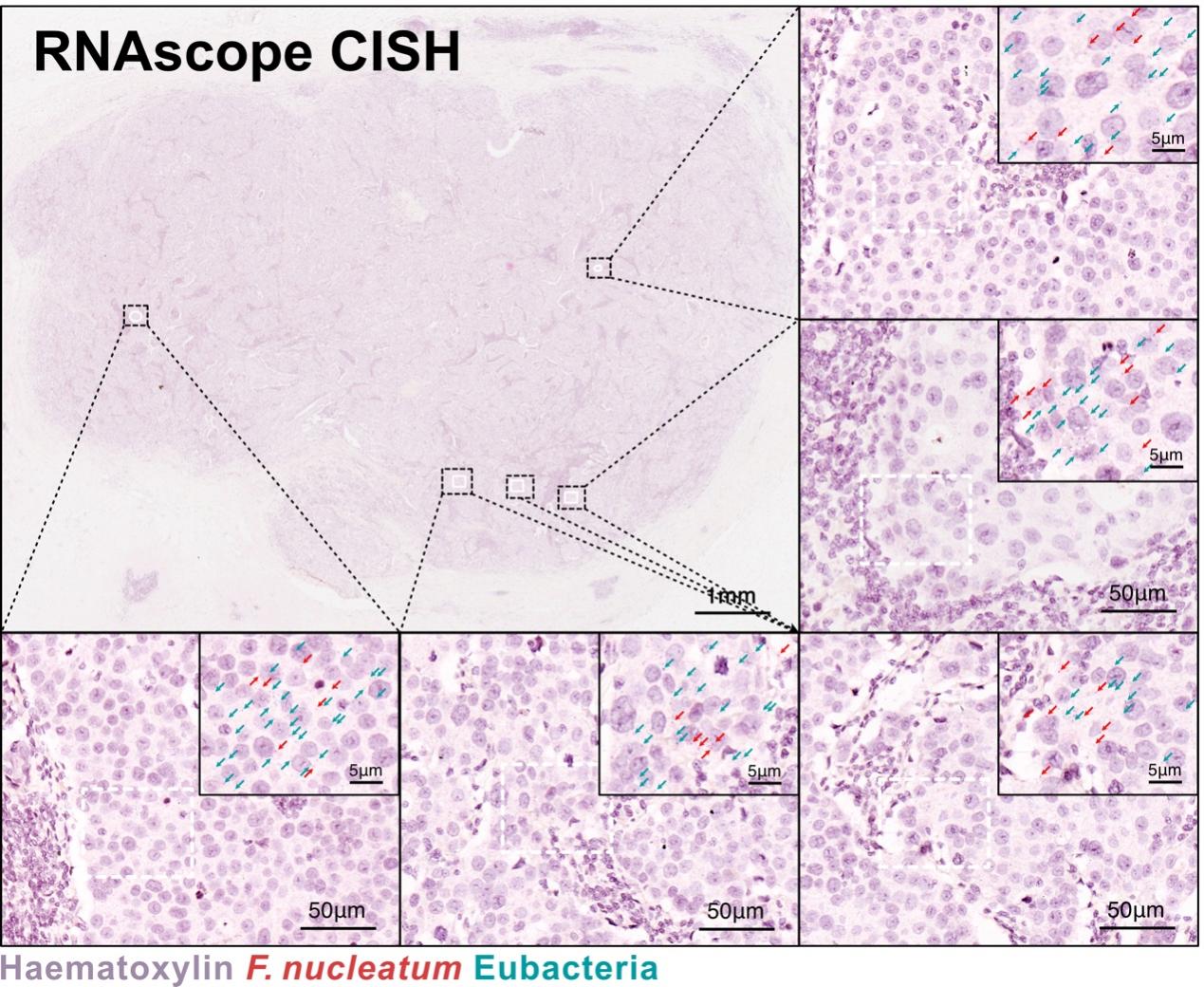


**Supplementary Figure S4: RNAscope-CISH images displaying the spatial distribution of bacteria in BC tumor tissue. The red signal highlights *F. nucleatum*, while the blue signal marks Eubacteria (universal bacterial probe)**


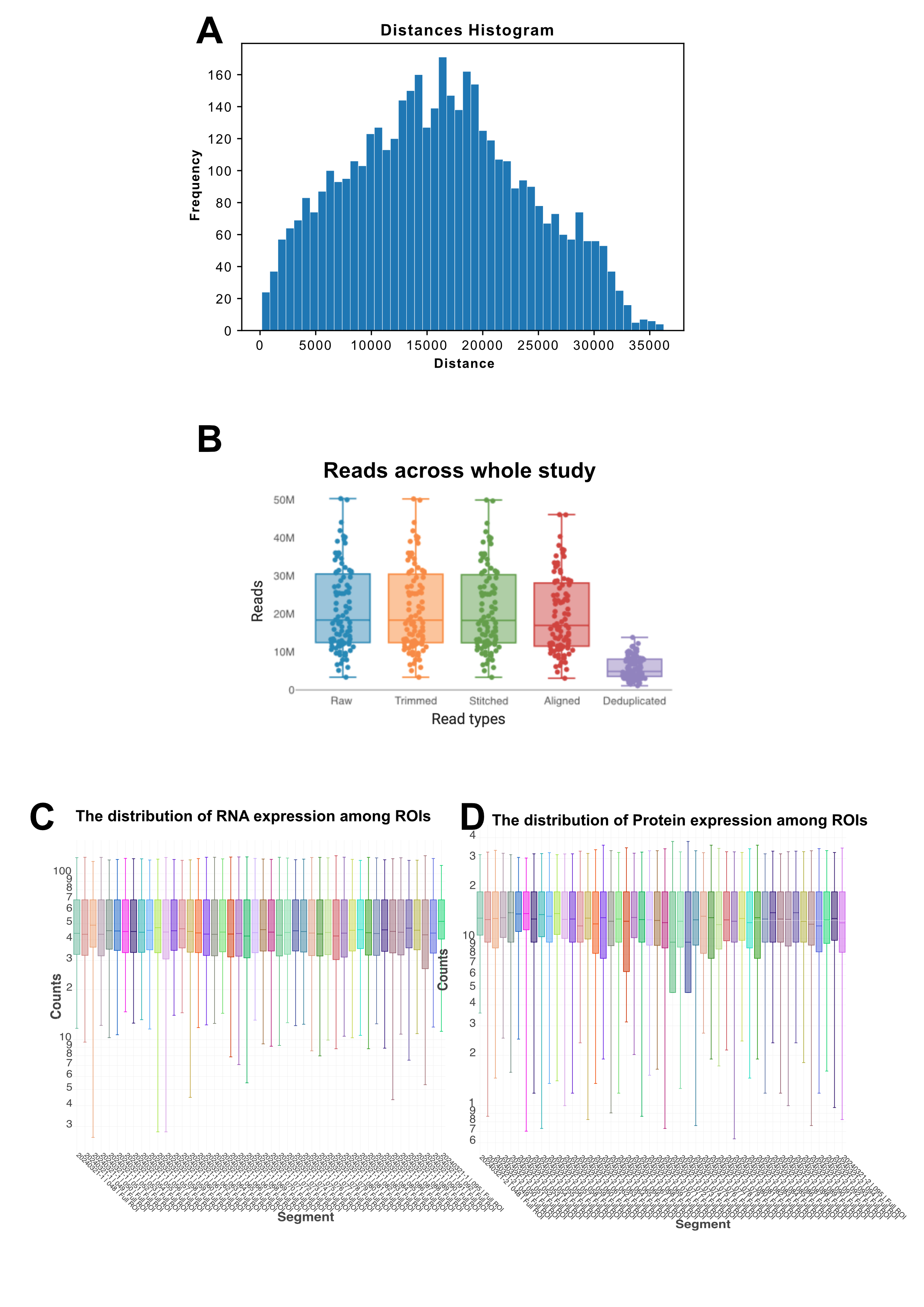


**Supplementary Figure S5: GeoMx DSP spatial multi-omics experiments**


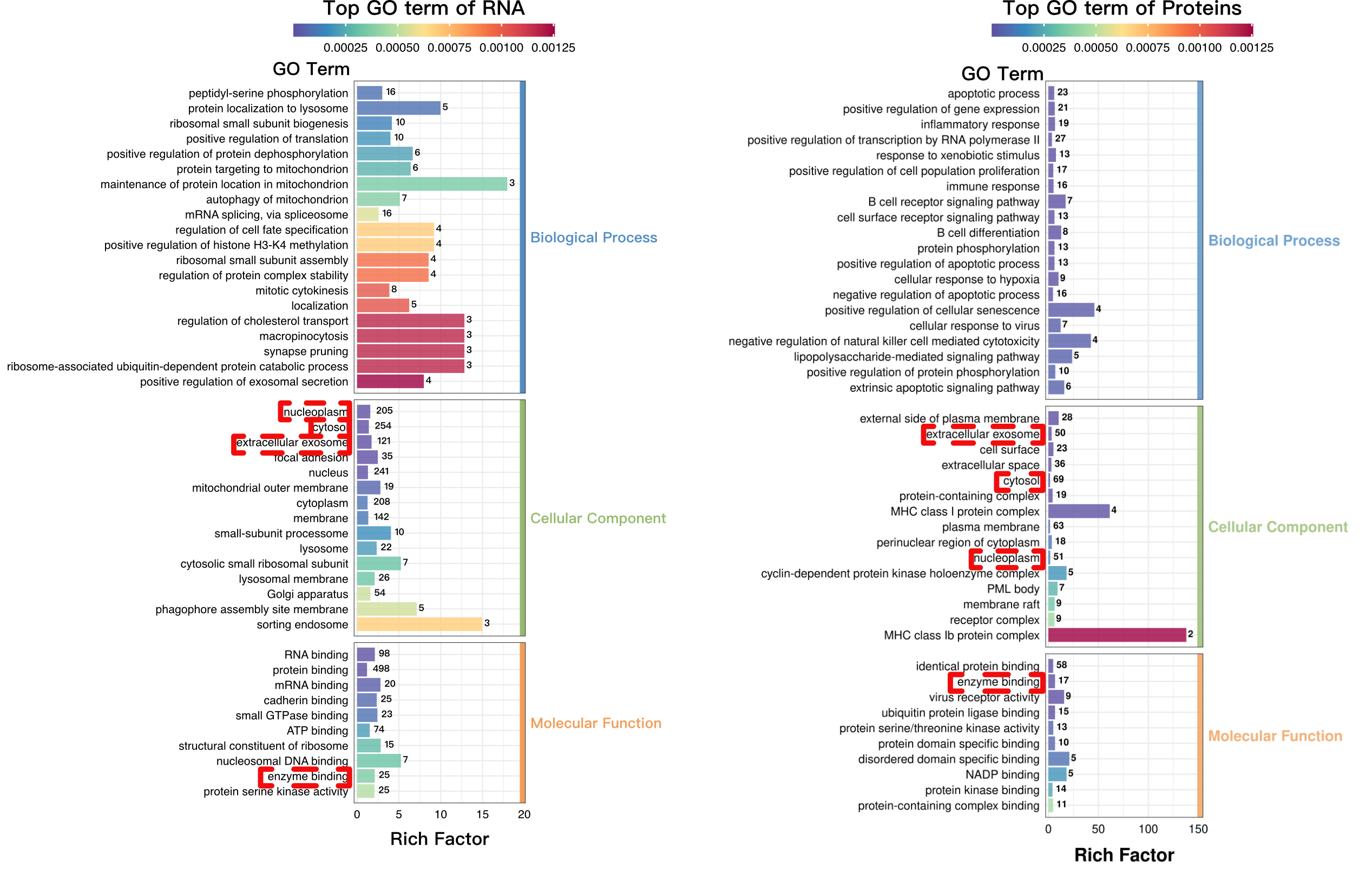


B

A

**Supplementary Figure S6:** GO enrichment analyses of significantly different RNAs and proteins.


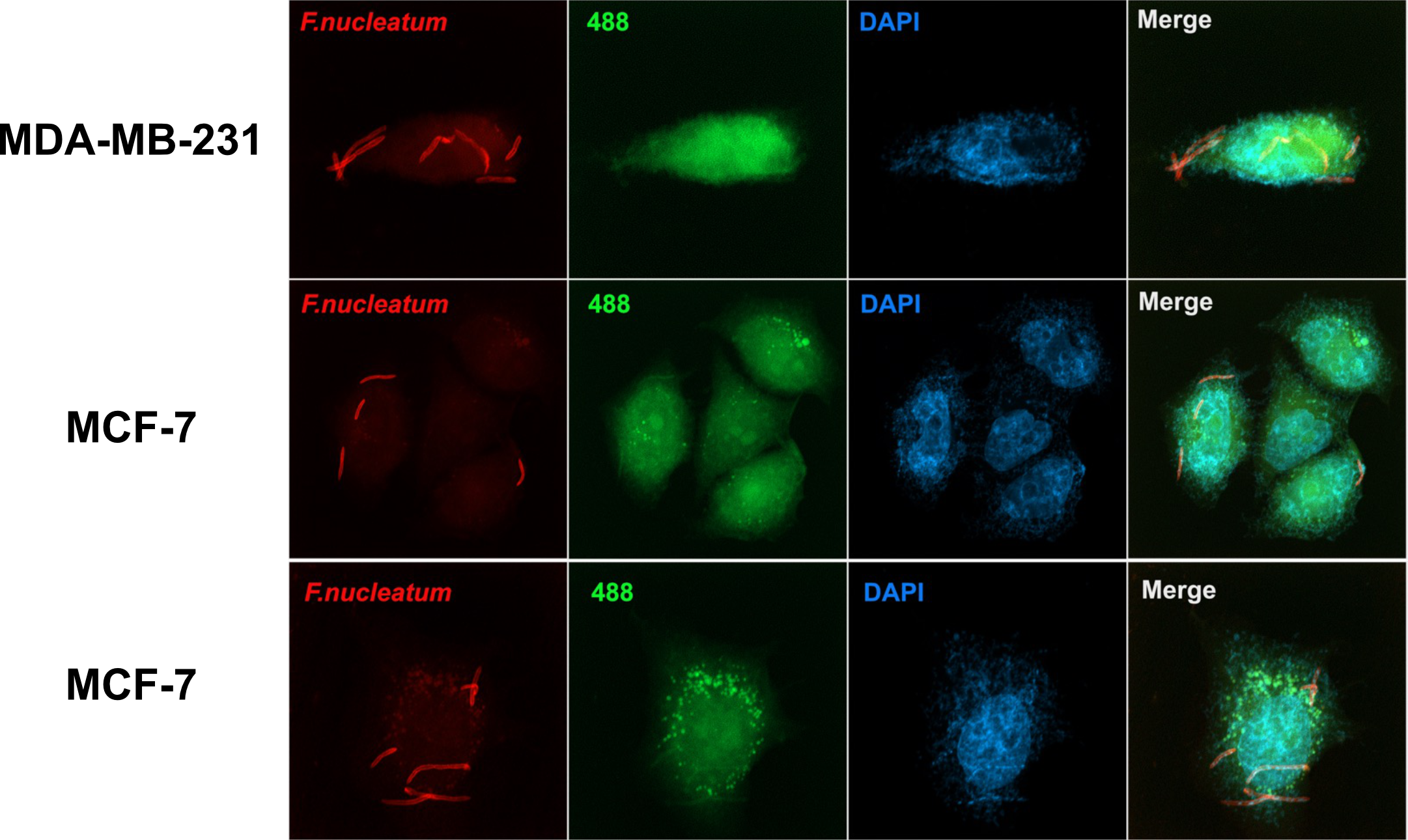


**Supplementary Figure S7: Confocal microscopy images.**


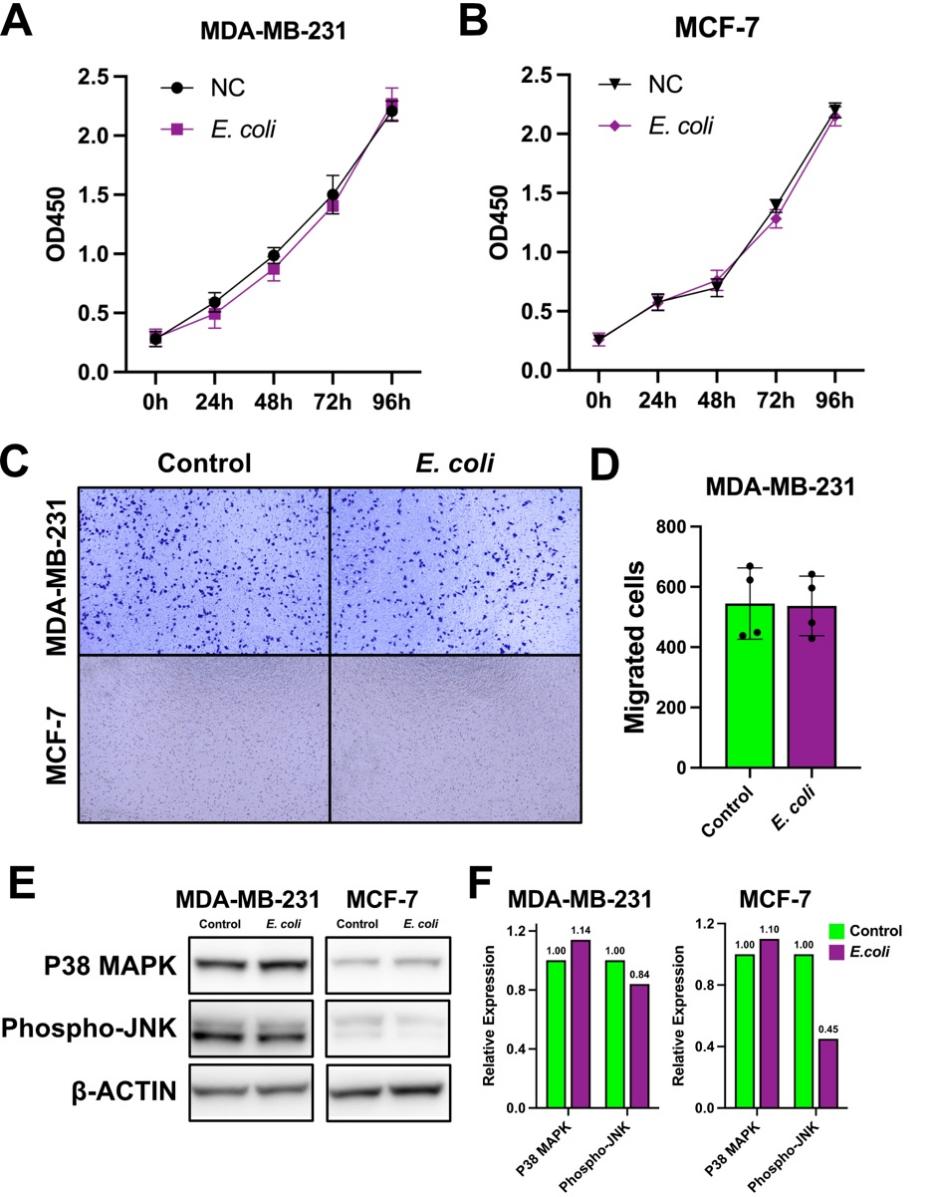


**Supplementaryary Figure S8: Effects of *E. coli* infection on the proliferation, migration, and MAPK signaling activation in breast cancer cell lines MDA-MB-231 and MCF-7.**


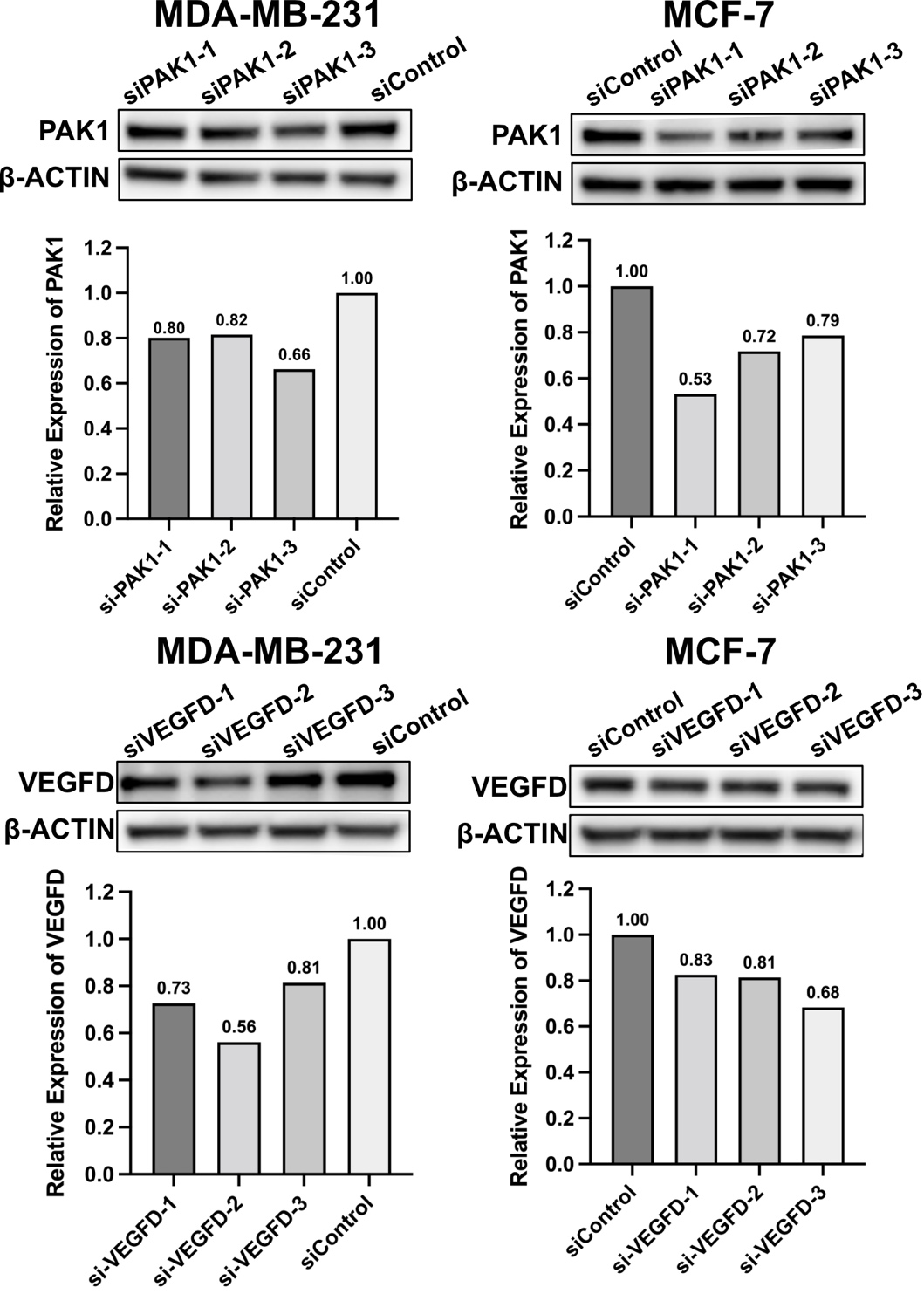


**Supplementary Figure S9: Western blotting (WB) results showed that VEGF and PAK1 protein expression was significantly reduced after siRNA interference.**
